# Behavior is movement only but how to interpret it? Problems and pitfalls in translational neuroscience—a 40-year experience

**DOI:** 10.3389/fnbeh.2022.958067

**Published:** 2022-10-05

**Authors:** Hans-Peter Lipp, David P. Wolfer

**Affiliations:** ^1^Institute of Evolutionary Medicine, University of Zürich, Zürich, Switzerland; ^2^Faculty of Medicine, Institute of Anatomy, University of Zürich, Zürich, Switzerland; ^3^Department of Health Sciences and Technology, Institute of Human Movement Sciences and Sport, ETH Zürich, Zürich, Switzerland

**Keywords:** comparative, neuroanatomy, neuroethology, behavioral testing, hippocampal lesions, hypothalamus, memory models, motor priming

## Abstract

Translational research in behavioral neuroscience seeks causes and remedies for human mental health problems in animals, following leads imposed by clinical research in psychiatry. This endeavor faces several problems because scientists must read and interpret animal movements to represent human perceptions, mood, and memory processes. Yet, it is still not known how mammalian brains bundle all these processes into a highly compressed motor output in the brain stem and spinal cord, but without that knowledge, translational research remains aimless. Based on some four decades of experience in the field, the article identifies sources of interpretation problems and illustrates typical translational pitfalls. (1) The sensory world of mice is different. Smell, hearing, and tactile whisker sensations dominate in rodents, while visual input is comparatively small. In humans, the relations are reversed. (2) Mouse and human brains are equated inappropriately: the association cortex makes up a large portion of the human neocortex, while it is relatively small in rodents. The predominant associative cortex in rodents is the hippocampus itself, orchestrating chiefly inputs from secondary sensorimotor areas and generating species-typical motor patterns that are not easily reconciled with putative human hippocampal functions. (3) Translational interpretation of studies of memory or emotionality often neglects the ecology of mice, an extremely small species surviving by freezing or flight reactions that do not need much cognitive processing. (4) Further misinterpretations arise from confounding neuronal properties with system properties, and from rigid mechanistic thinking unaware that many experimentally induced changes in the brain do partially reflect unpredictable compensatory plasticity. (5) Based on observing hippocampal lesion effects in mice indoors and outdoors, the article offers a simplistic general model of hippocampal functions in relation to hypothalamic input and output, placing hypothalamus and the supraspinal motor system at the top of a cerebral hierarchy. (6) Many translational problems could be avoided by inclusion of simple species-typical behaviors as end-points comparable to human cognitive or executive processing, and to rely more on artificial intelligence for recognizing patterns not classifiable by traditional psychological concepts.

## Introduction

### The problem

Translational research extrapolates findings from basic research in animals to clinical fields. On the other side, clinical scientists are supposed to stimulate basic scientists to focus on research topics that may result faster in clinical and therapeutical solutions (Sampath and Ramchandran, 2014). Translational approaches in many fields of medicine are based on a solid body of biochemical, histological, and molecular knowledge of different organs in both standard laboratory animals and human tissue. Most of that knowledge reflects evolutionarily conserved features and processes that are unlikely to vary strongly between species. With respect to the brain, this also includes cellular and molecular processes in and between neurons and glial cells. Thus, research requests by clinicians focusing on such topics are often feasible and realization mostly depends on available resources and motivation. The situation is different in behavioral neuroscience. For one, there is a larger group of neurobehavioral scientists and animal psychologists without clinical goals but presenting their approaches as clinically meaningful, often by investigating genetically modified mice. On the clinical side, neuropsychologists, psychotherapists, and psychiatrists are requesting a focus on basic research eventually solving their problems but are facing an immense number of articles reporting that genetic targeting of mechanisms at the cellular level in mice and rats somehow produces behavioral changes that may or may not fit into clinical symptoms.

The main problem is simple. After some 200 years of brain research in mammalian species and humans, there is no commonly accepted view of how the brain operates as a whole in different species. There is no doubt that the structure of neurons, axons, synapses, and related molecular processes is vastly similar. But how to combine these components to reach an easy understanding of behavioral species differences–the *condition sine qua non* in translational research–has remained frustratingly elusive. The problem is compounded by the ill-defined term “behavior” which seems to have numerous connotations and interpretations even in neuroscience. However, in strict terms, the only output of the brain is the control of striate and smooth muscle fibers. Movements caused by striated muscles can be observed, while many effects on physiology are mediated by regulation of blood flow caused by the action of smooth muscle fibers on small arteries. Therefore, the sole and only purpose of the nervous system in all autonomously moving species is to produce patterned movements and homeostatic co-regulation of the milieu interieur. Evolution has shown an increasing investment in myelinated long fiber tracts enabling the brain to fine-tune motor acts (Mota et al., 2019) and everyone who has ever dissected the massive corticopontine fiber tracts in the human brain can only agree. However, the view of a brain shaped by its motor output structures is unpopular in the field of behavioral science but should be at least understood by translational scientists.

### Two brands of neuroscientists

Neuroanatomists and neuroethologists are easier to convince since they have a rather straightforward view: above the level of the hypothalamus, mammalian brains have a rather uniform design. The thalamus and its ascending fibers distribute radially, like colored laser beams in a nightclub, various subcortical and sensory inputs to a dome-like screen organized in columns (Supplementary Figure S1). In contrast to a nightclub, that screen is heavily interconnected, permitting spreading and blending of patches of excitation yet primarily to neighbored columns. But the basic design of the neocortex is radial (Rakic, 2009) and not tangential, being driven by thalamic output that remains topographically arranged with little crosstalk between thalamic channels. The output of the mammalian neocortex is equally radially organized: myelinated fibers converge eventually to the mesencephalic midbrain and pyramidal tract handling together the precise activation and sequencing of striated muscle cells. Thus, the output of the entire brain is squeezed and twisted into a somatotopically arranged pattern of axons known as “homunculus”, “simiaculus”, or “musculus” depending on the species. Obviously, the contribution of the neocortex is shaped somehow by iteratively passing through thalamus, basal ganglia, and cerebellum in the form of aligned channels. But how the neocortex achieves the cognition-based selection and fine-tuning of output channels is beyond the neuroanatomist's experience and scope and thus left to others.

These others include a variety of scientists from fields, such as neuropsychology, psychiatry, and system theory, that have a penchant to perceive the cortex from its sensory side, preferably visual. Other neuroscientists focus on specific functional features resulting in tags and labels, such as “emotional brain”, “visceral brain”, “social brain”, “mind”, and “consciousness”, to name a few. Such approaches are mostly theory-driven and try to read the observed motor patterns in animals according to predictions made by psychological theories. But since all types of behaviors including complex patterns of movement, such as language and writing, are lastly motor endpoints, behavioral neuroscientists face the same problem as neuroanatomists in explaining the fine-tuning of the so-called executive functions. In principle, behavioral research in animals resembles interpreting the interiors of a moving black box and becomes increasingly tentative with evolutionary distance. At least in humans, advances in whole brain functional imaging and large-scale electrical patterns including EEG and event-related potentials (ERP) provide guidance, increasingly also in animals. However, for technical and conceptual reasons, the focus of these techniques is primarily on the cortex, while visualizing subcortical activity patterns is more difficult and demanding. Given this constellation, the task of translational neuroscientists is doubly difficult. They must cope with the situation of lacking fundamental knowledge of how cognition translates into motor pattern in mammals, but they are supposed to use animals, analyze their movements, and find critical brain mechanisms and processes that permit to realize clinically defined goals. Another difficulty is the flood of genetically modified mice in which genes theoretically meaningful for the proper functioning of memory and emotions were altered but the resulting changes in motor patterning of the transgenic animals appear frustratingly similar: either the mice move too fast, too slow, too early, or too late.

### An overview of the article

Therefore, the goal of this article is to provide concepts and personal insights enabling scientists to tackle problems inherent in behavioral translational analysis and to avoid pitfalls in interpretation and experimental approaches. Concepts and insights are derived from 40 years of experience in various fields of behavioral neuroscience, often working with natural and genetically induced variations of brain and behavior in mice but also with several other mammalian and avian species. The article will thus review the older work of the authors but also present unpublished data bolstering some of the claims. Since both, reasonable knowledge of comparative neuroanatomy and neuroethology, are necessary for a translational approach, a condensed overview of brain architecture related to behavior and cognition will be given first. A discussion of hidden species differences in neuroanatomy of mice and humans follows, focusing in some detail on the hippocampus and its integration into the cortical functional architecture. The next section is devoted to key elements of mouse behavior and its role in daily life and evolution. As mechanistic explanations are often requested in translational research, examples from own research will show that unpredictable events in brain plasticity occur more frequently than thought and should be integrated conceptually. This is joined by a functional analysis of hippocampal lesions in mice as observed in the laboratory and under semi-naturalistic conditions. Finally, the article suggests that placing the basal forebrain structures and the supraspinal motor system (SMS) at the top of the brain hierarchy facilitates conceptually translational approaches and provides concrete suggestions and advice for experimental strategies in the field.

## Neuroanatomical differences and similarities between mice and humans

### Sensory systems

**Figure 1:**
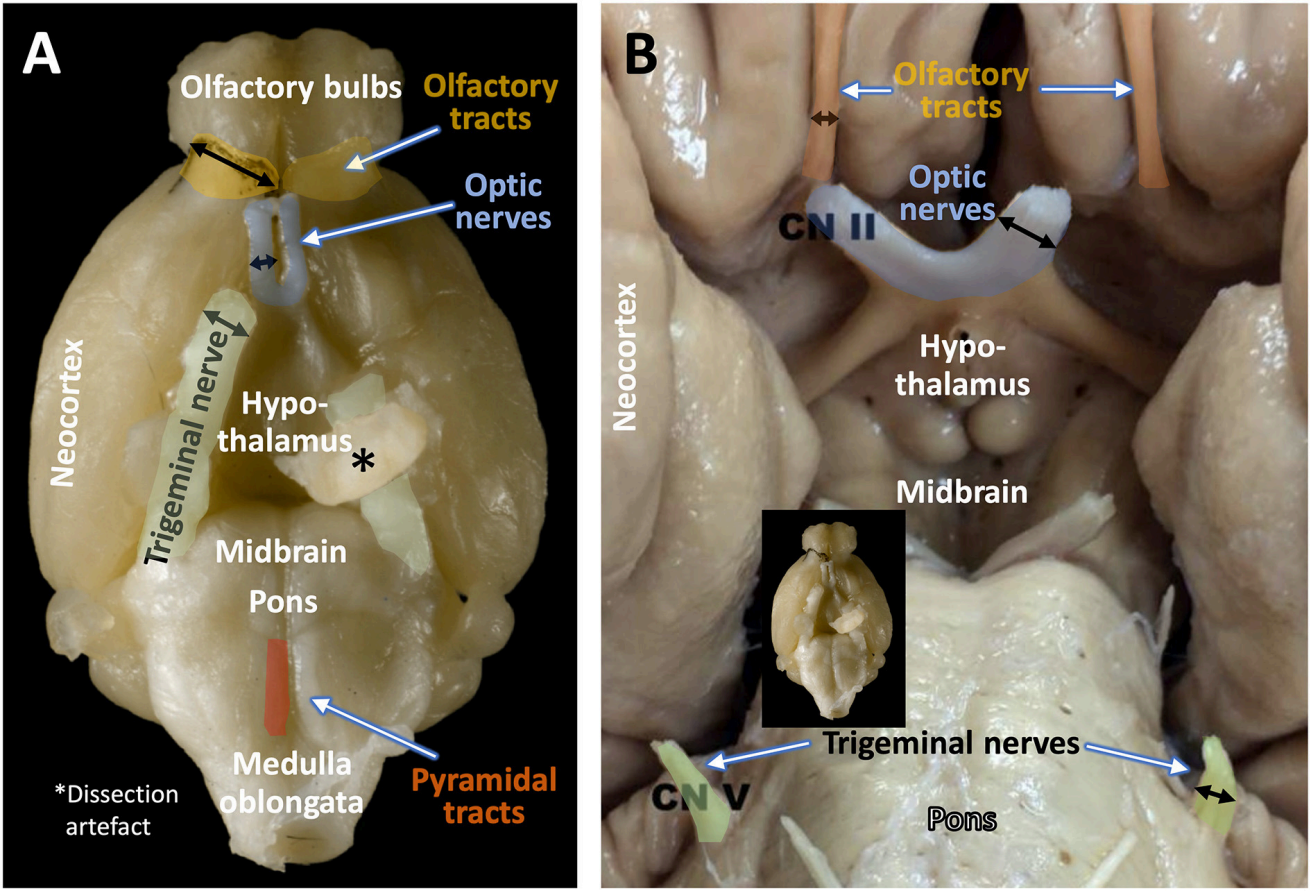
Size relations of olfactory tract, optic nerve, and trigeminal nerve in the mouse **(A)** and the human **(B)**. Black arrows indicate the relative diameter of the respective fiber bundles. Note the predominance of olfactory and trigeminal input over visual input in the mouse, and the reverse situation in the human brain. The original image of the mouse brain was kindly provided by H.J. Schröder, University of Cologne, and the copyright is with Springer Nature Switzerland AG, Neuroanatomy of the Mouse, 2020 (Schröder et al., 2020). The image of the human brain (Sonne and Lopez-Ojeda, 2022) is downloadable from Sonne J, Lopez-Ojeda W. Neuroanatomy, Cranial Nerve. [Updated 2021 Nov 14] under the Creative Commons Attribution 4.0 International License (http://creativecommons.org/licenses/by/4.0/) and has been re-labeled by the authors.

Species-differences in sensory capacities can easily be identified in Figure 1 showing the base of the brain with the main cranial nerves. In humans, the optic nerve and oculomotor nerve dominate in volume, while both olfactory tract and trigeminal nerve are comparatively small. The mouse, however, shows voluminous olfactory tracts yet small optic nerves. In comparison to humans, the mouse trigeminal nerve is also much larger. Whether the cochlear (auditory nerves) differ comparatively in size cannot be judged by such ventral views, but even the large ears of mice as compared to the ones of rats imply the importance of auditory cues for the mouse. With the exclusion of olfactory inputs, all these sensory modalities are channeled through the thalamus to the neocortex. There, the sizes of sensory cortical fields correspond to the sizes of the cranial nerves, specifically in the barrel cortex in mice that dominates auditory and visual fields (Froudarakis et al., 2019). Nonetheless, a literature search of mouse learning in relation to sensory abilities of specialized cortex regions reveals about 10 articles for vibrissae-based learning, and some 1,500 articles involving the visual cortex. Given the importance of whiskers for mice and their propensity of living in poorly illuminated environments, this represents an investigative mismatch. In fact, mice can learn vibrisso-tactile discrimination tasks quite easily (Warren et al., 2021), the problem is to analyze their learning strategies and type of errors (Lipp and Van der Loos, 1991). For interpreting mouse behavior correctly, scientists should try to imagine a world of smells (an “olfactorama” sensu Rekow et al., 2022) and to envision what it means to move around with a set of whiskers that extend farther than the reach of the hands. But the terms “imagine” and “envision” reveal the strong visual bias of humans. To be fair, the importance of olfaction for learning in mice is well documented but the translational value is difficult to estimate, not at least because the performance of human olfactory system is certainly inferior to one of many non-primate species.

### Structural layout of the mammalian brain

#### The “old” brain and its connections

The structural layout of neuronal groups and fiber connections in basal forebrain and upper brainstem appears rather similar across vertebrate species, as it corresponds to the phylogenetically conserved “minimal brain” or “old brain” able to maintain sufficiently sophisticated motor outputs to ensure survival in various ecological niches. The basic layout and design of the vertebrate brain with a central ventricular system and a radial structure formed by glial and neuronal cells may have a history of 560 Mio years (Suryanarayana et al., 2020). The origin of the modern mammalian multilayered cortex interconnected by the corpus callosum dates back probably no further than 120 million years (Mihrshahi, 2006).

In terms of behavioral control, the most powerful structure is the phylogenetic old motor control system, the supraspinal motor system (SMS) in the reticular formation, and the rostral brain stem. An incredibly complex set of nuclei and axonal connections mediates species-specific behavior (ethological “elements”) and automated behavioral routines such as locomotion and integrates them with “pyramidal” input from the neocortex (Esposito and Arber, 2016; Josset et al., 2018). The SMS is bidirectionally connected with phylogenetic old sensory structures such as superior colliculus for visual inputs and inferior colliculus for auditory input. The other inputs to the SMS originate from the basal forebrain system (BFS) including anterior olfactory, septal nuclei, and the hypothalamic area by means of short reciprocal connections forming the medial forebrain bundle (MFB) and the dorsal longitudinal fascicle (DLF). The hypothalamic area is also extremely complex, its nine nuclei integrating physiological, humoral, motivational, and limbic inputs, often in set-points regulating homeostatic processes (Dougherty, 2020). From a behavioral point of view, the connections of the hypothalamus to the SMS orchestrate flexibly species-specific behaviors. For example, electric stimulation of the ventromedial hypothalamus (VMH) in marmoset monkeys elicits defensive vocal threat (chattering) and slashes toward a cage companion, but in presence of a fear-inducing observer the stimulated monkey switches to mobbing behavior, a species-specific behavior including lateral body swaying and sharp mobbing calls (Lipp, 1978; Lipp and Hunsperger, 1978). Such behavioral flexibility is regularly observed when stimulating hypothalamic areas in other species (Lipp, 1979; Halasz et al., 2002; Haller, 2013; Kruk, 2014; Wang et al., 2021; Bang et al., 2022) and appears to be a species-invariant feature.

#### The neocortical sensorimotor loop system

**Figure 2:**
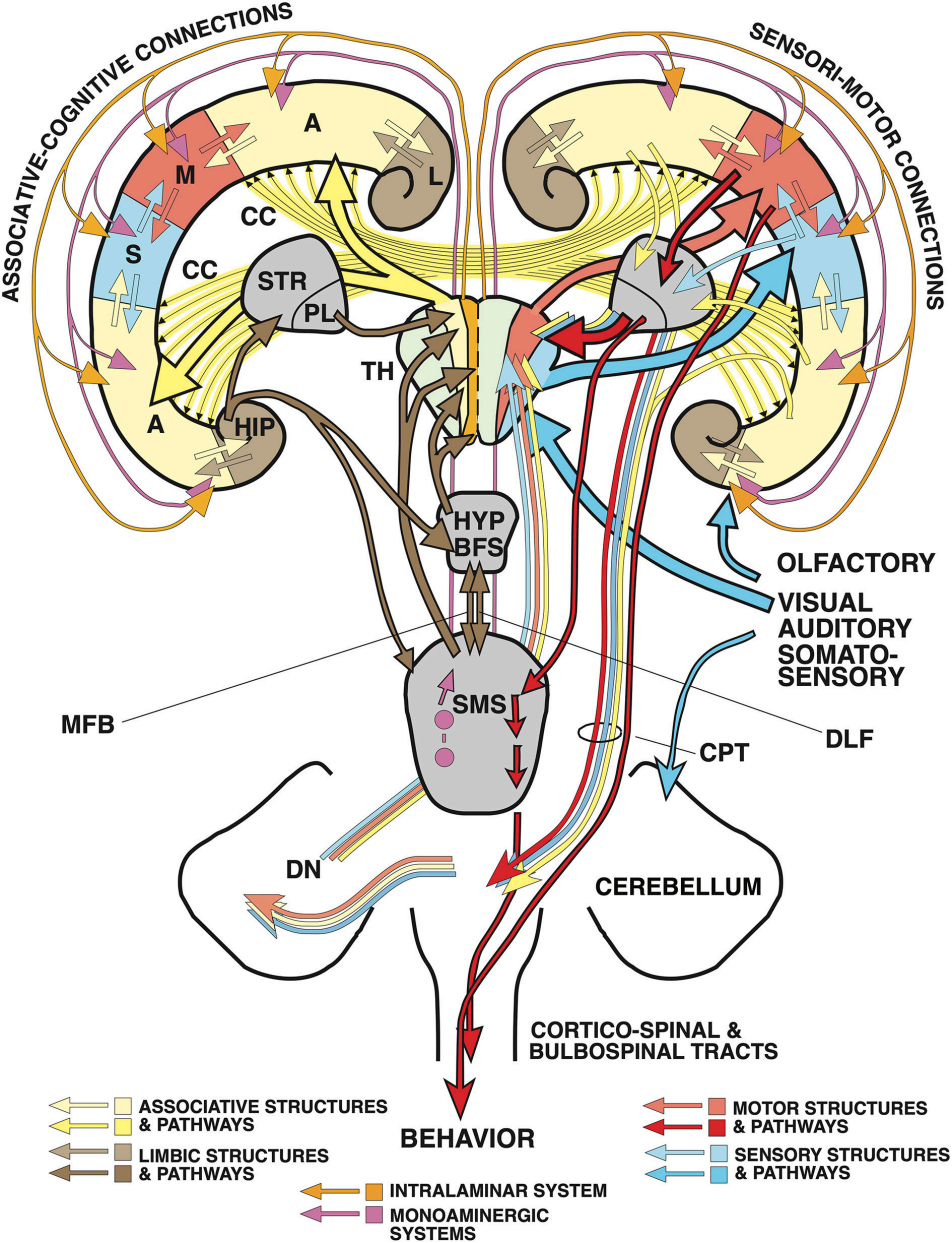
“Cognitive” and “non-cognitive” circuitry of the mammalian brain. At right, the main connections of the primary sensory (S) and motor cortex areas (M) are described in further detail in the text, see also Lipp and Wolfer (1998). The left side illustrates the connectivity of the “cognitive” (associative and limbic) cortex. Principal inputs to association cortex (A) originate in the hypothalamus (HYP) and the reticular formation (RF) and reaches the non-specific (associational) thalamus (TH, yellow) from where ascending divergent fibers reach many parts of the associative cortex (A). The connections of the associative cortex include reciprocal fiber connections with neighboring areas subserving tangential spread of information. Part of the output of the association cortex is fed into the motor fine-tuning loops, to the other hemisphere *via* the corpus callosum (CC), and *via* polysynaptic chains into the (marginal) limbic cortex (L) and the hippocampal formation (HIP). The cingular and entorhinal cortex send also fibers to the limbic basal ganglia (STR) where the limbic pallidum (PL) sends (probably inhibitory) fibers to the intralaminar system of the thalamus and to the anterior thalamic nuclei. Other fibers from hippocampus and amygdala reach the hypothalamus and reticular formation, both structures sending fibers to the associative thalamus from which divergent axons spread again to the associative cortex portions. Thus, the activity of the entire cortex can be structured through ascending control systems: the intralaminar system (orange), and the ascending monoaminergic systems (purple), see also text. Any alteration in these comparatively minor structures, be it genetic, developmental, or pathological, has thus a potentially powerful impact. An earlier version of this figure has been published by Lipp and Wolfer (1998), the actual one is modified and has been re-labeled by H.P.L.

The thalamus distributes afferent sensory inputs in diverging bundles creating somatotopic, retinotopic, or cochleotopic maps in primary sensory and motor cortex areas that show, except for auditory input, few, if any, callosal projections (Figure 2). Their activity patterns blend with the adjacent association cortex and transform eventually into the marginal limbic cortex and hippocampus. The motor cortex gives origin to the well-known somatotopic corticospinal (pyramidal tract)—the only efferent system of the neocortex passing to the spinal cord—which mediates fine-tuned movements. The output activity of the motor and premotor cortex is constantly refined by two loop systems: a topographically mapped (but only partially somatotopically organized) feedback loop from all cortical areas descends as corticopontine tract to the pontine nuclei, from where cerebellar mossy fibers ascend to the contralateral cerebellum. The ponto-cerebellum integrates polymodal neocortical activity patterns with proprioceptive somatotopic inputs from the vestibulo- and spinocerebellum, and bundles converging fibers to the deep cerebellar nuclei (DN). These send *somatotopically* arranged fibers chiefly to the motor thalamic areas. The other topographically ordered loops include polymodal fibers from most cortical regions converging on the striatum (STR) from where inhibitory fibers of the pallidum (PL) reach the motor thalamic nuclei where they control the ascending throughput of the cortico-ponto-cerebello-thalamo-cortical loop. Note the distinction between somatotopic (just mapping a motor “homunculus”) and polymodal yet topographically organized projections (keeping fibers from specific cortex regions together) influencing the refinement of the motor output. In simple terms, the total activity pattern of the neocortex is being blended, twisted, and forced to drive the motor “homunculus” in spinal cord and brain stem, or, for that matter, a corresponding “simiaculus” in monkeys or “musculus” in mice.

#### The “cognitive” loop system

The other loops as shown in the left half of Figure 2 depict the arrangement of the “thinking brain” (Cook, 1986). As with the posteriorly located somatosensory inputs, ascending fiber tracts from the thalamus diverge radially to reach a variety of more frontally located cortical areas including various types of associative cortex fields, not unlike a bouquet of flowers in the form of neocortical columns. However, these remain connected with proximal ipsilateral columns but also with near-symmetrically located contralateral columns on the other side. The main difference to the sensorimotor cortex is that the thalamic input originates from the midbrain and from a chain of interconnected structures of the basal forebrain, such as the anterior olfactory nuclei, the nucleus accumbens septi, the septum, and from the various divisions of the hypothalamus. Thus, the associative cortex is not only concerned with handling the interactions with cortical regions but is driven substantially by motivationally relevant structures. Given that the thalamocortical fibers of the limbic and “non-specific” thalamus ascend bilaterally, their impact on a given neocortical column appears even stronger than the input from neighbored ipsilateral columns alone, because of the corpus callosum connecting near-homotopic cortical fields. Part of the output of the association cortex is fed into the motor fine-tuning loops. Another portion reaches *via* polysynaptic chains the (marginal) limbic cortex and the hippocampal formation, but this pathway for propagation narrows considerably (see also **Figure 4**). The cingular and entorhinal cortex also innervate the ventralmost basal ganglia, including the nucleus accumbens and the ventral pallidum, the later inhibiting the anterior thalamic nuclei and possibly the intralaminar system of the thalamus. Other fibers from hippocampus and amygdala reach the hypothalamus and reticular formation, from where fibers ascend again to the associative thalamus, closing a cortico-limbic-hypothalamo-thalamo-cortical loop. Thus, the general layout of the associative cortex and its subcortical loops resembles the sensorimotor portions of the neocortex.

Taken together, the integrative activity of the entire cortex is structured by the thalamus (Shine, 2021): non-specific nuclei drive associative and limbic cortex, the strength of driving being regulated by intrathalamic inhibition of throughput channels. For one, tonically active inhibitory fibers from the limbic pallidum regulate the transmission of subcortical input to the anterior thalamic nuclei that are connected to prefrontal and limbic areas. In addition, the second GABAergic intrathalamic system, the reticular nuclei (Crabtree, 2018), appears to receive input from the rostral reticular formation and the SMSs that could throttle or amplify any sensory and associative input to the neocortex. Finally, the thalamus harbors the intralaminar system (a powerful input amplifier of widespread cortical regions). Further ascending control systems in the brain stem are the aminergic projections (dopaminergic, noradrenergic, serotoninergic) which innervate the neocortex in a less precise fashion than topographically ordered projections. Any alteration in these comparatively minor structures, be it genetic, developmental, or pathological, has thus a potentially powerful impact on neocortical activity.

## Focus on the hippocampus

In evolutionary terms, the hippocampus is a relative newcomer as the classic trisynaptic loop with mossy fibers is found in mammals only (Witter et al., 2017). In less encephalized species, it appears that the hippocampus is occasionally the largest associative cortical structure as nicely observed in the African elephant shrew (*Elephantulus myurus)* whose hemispheres appear to consist primarily of hippocampi yet have only small neocortical areas (Slomianka et al., 2013). Nonetheless, it is perfectly adapted to its habitat and its rodent family is found widely across Subsaharan Africa.

### Hippocampal functions: History of concepts underlying translational research

Functionally, the hippocampus proper was considered for a long time a somewhat enigmatic structure. Early lesion studies listed by O'Keefe and Nadel (1978) revealed a bewildering variety of behavioral effects including deficits in some species-typical behaviors (Kim et al., 1970, 1971) yet often contradictory results with respect to acquisition and performance in learning paradigms used in experimental psychology. Likewise, electrical stimulation of the hippocampus provoked chiefly brief attentional responses, much in contrast to the spectacular behaviors elicited by electrical brain stimulation in the hypothalamus and the amygdala. Since many lesion studies entailed hyperactivity, the consensus was that the hippocampus appeared to have a generally inhibiting role for spontaneous locomotor behaviors (Douglas, 1967; Simonov, 1974), but why and how remained unclear.

**Figure 3:**
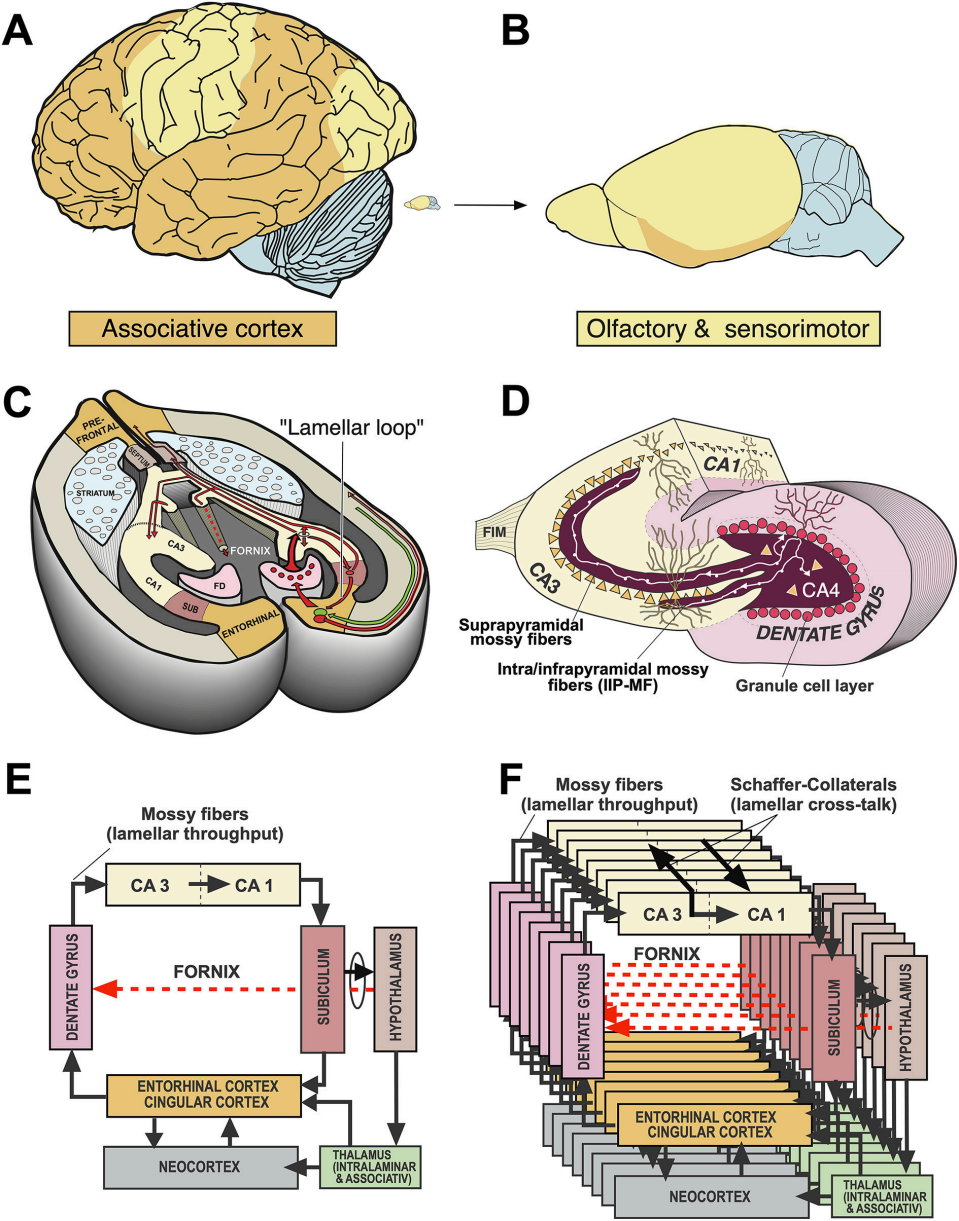
Comparative aspects of hippocampal circuitry. **(A)** In humans, a huge part of the neocortex is devoted to associative cortex, while cortical regions with specialized sensory or motor function are relatively restricted. **(B)** The rat neocortex includes mostly specialized sensorimotor areas but also olfactory input. Associative cortex is reduced and appears marginal. **(C)** The horizontal section through a mouse brain reveals that the associative cortices fold in to form a relatively large hippocampal formation that is topped at its end by the (late developing) dentate gyrus. The right half shows the typical feed-forward loop originating from the entorhinal cortex that transmits topographically ordered input (green arrow) from the remaining associative cortex as perforant path to the granule cells (red) in the dentate gyrus. From there, mossy fibers run along the pyramidal cells of hippocampal subregion CA3. The targeted neurons in CA3 give off axonal branches known as Schaffer collaterals that transmit the inputs to CA1 and eventually to the subiculum. The subiculum gives collaterals to the entorhinal cortex, closing the so-called lamellar loop, but is also sending fibers *via* the fornix that end in various subcortical structures, such as septum and hypothalamus. Note that the fornix contains also ascending fiber projections (see below). While the lamellar loop appears similarly organized in both species, the rodent hippocampus shows massive commissural projections largely lacking in humans in which commissures connect the entorhinal cortices. **(D)** 3D-scheme of the hippocampal mossy fiber projections. Axons of the granule cells run along the CA3 region contacting numerous pyramidal cells with so-called giant boutons. **(E)** Planar scheme of the lamellar (“trisynaptic”) loop starting at the entorhinal cortex, with the subiculum as the main output structure to both cortical and (*via* Fornix) to subcortical structures along the basal forebrain. The red arrow symbolizes the ascending projection from the hypothalamus to the hippocampus (for details see Figure 13B). **(F)** Stacked trisynaptic lamellae showing the lateral spread of the Schaffer collaterals, probably less in CA3 than in CA1. FD, fascia dentata (dentate gyrus); FIM, fimbria hippocampi; SUB, subiculum.

Significant progress resulted from an in-depth neurophysiological analysis of hippocampal circuitry by the Andersen group. They described the now classical “trisynaptic” feed-forward loop (Andersen, 1959; Andersen et al., 1973), the lamellar organization of the hippocampus (Andersen et al., 1969) as shown in Figures 3, **15**, the phenomenon of long-term potentiation, LTP (Bliss and Lomo, 1973) and the *in-vitro* preparation of hippocampal slices (Skrede and Westgaard, 1971). Taken together, this permitted a vision of the hippocampus in which various inputs could be held temporarily in functionally separate channels in CA3 yet permitted to interact by means of Schaffer collaterals. This vision has been criticized, e.g., by Buszaki (Lisman et al., 2017) but remains essential for most theories relying on functional mapping of cortical and subcortical connections in the hippocampus (Andersen et al., 2000; Sloviter and Lomo, 2012). Eventually, two main lines of interpretation of hippocampal function emerged, one considering the hippocampus as coordinating ongoing motor behavior, the other postulating hippocampo–neocortical interactions based on theoretical concepts.

The most straightforward interpretation was offered by Vanderwolf who discovered the septohippocampal theta rhythm (Vanderwolf, 1969) and perceived the hippocampus as a machinery adapting olfactory inputs to movement-related proprioception (Vanderwolf, 2001). Most animals rely on smell to set goals that must be either approached or avoided, and the hippocampus is continually balancing such approach–avoidance conflicts. While this notion was deemed too simplistic by many, one should note that even recent MRI studies in human hippocampi have visualized approach–avoidance conflicts not explainable by cognitive theories (O'Neil et al., 2015). Thus, reading a summary of Vanderwolf (2007) view might be instructive for students of behavioral neuroscience, including his (unsuccessful) cautioning against mixing empirical neuroscience with antique philosophical ideas. The second theory of the hippocampus as modulator of conflicting behavioral tendencies was established by Gray and McNaughton (1982) who argued that this primordial hippocampal function is overshadowing potential memory and cognitive processes, whose significance for ongoing adaptation of behavior must be demonstrated experimentally using the sophisticated toolbox of animal learning psychology (Davidson and Jarrard, 2004). Yet, as explained before, the way how ongoing behavior is shaped by “cognitive” structures is still not known, and many of the behavioral results reviewed and reported here still fit the views of Vanderwolf and Gray better than other theories based on hippocampo–neocortical interactions.

The theoretical foundations of the main “cognitive” explanations of hippocampal function can be tracked back to Marr (1971) and O'Keefe and Nadel (1978). In essence, they rely heavily on single-cell neurophysiological ideas and techniques. Marr's computational approach perceived the hippocampal circuitry as holding temporarily a reduced assembly of daily neocortical activity and feeding it back during the night for permanent memory storage (Willshaw et al., 2015). His ideas also promoted the view of the hippocampus as an auto-associative network digesting neocortical input by completing or separating patterns of hippocampal activity thought to result from recurrent collaterals of CA3 and CA1 neurons (Yassa and Stark, 2011). The second and conceptually most influential approach was the monumental effort of O'Keefe and Nadel (1978) to overturn earlier views of hippocampal functions by successfully creating the notion that the hippocampus was sort of a neuronal map handling pieces and bits of ongoing neocortical activity to create a virtual Euclidean space supporting memory and guiding future actions. The theory gained support from neurophysiological studies showing the presence of rodent “place cells” firing in given locations but needed lengthy and not always convincing argumentation to refute interpretation of hippocampal lesion studies in terms of behavioral inhibition. Likewise, the cognitive map theory as presented initially barely tried to explain deficits in species-typical behaviors such as hoarding or circadian activity, a problem recognized by Nadel himself (Nadel, 1995).

### Rodent and human hippocampus: Similarities and differences

Macroscopically, rodents show smaller association cortices than more encephalized species, but the in-folded hippocampi represent still a substantial portion of the hemispheres (Figures 3A,B). This is in contrast to the human brain in which a dissected hippocampus has about the size of a thumb, weighs about 10 g (Patzke et al., 2014), and occupies only part of the temporal lobe. Morphologically, there are other clear differences. For one, the rodent hippocampus has extensive contralateral connections passing through the ventral hippocampal commissure, also known as associational/commissural pathway. In contrast, the human hippocampus proper has few commissural fibers. If any, they pass through the dorsal hippocampal commissure (the psalterium dorsale located below the corpus callosum) and play a minor role as judged by the volume of visible fibers. Thus, the human hippocampus appears to communicate chiefly with the ipsilateral hemisphere, being strongly lateralized itself as commissural interactions must take place *via* the commissural fibers of entorhinal and cingular cortex. Therefore, translational research in rodents must expect immediate contralateral responses to manipulations in one of the hippocampi that will be less or not observable in humans.

#### Species similarities in hippocampal architecture and hypothalamic projections

The general architecture of the hippocampus appears comparable in many species and is summarized in Figures 3C–F showing the well-known trisynaptic loop, labeled as “lamellar”, loop because it seems debatable how many stations that loop might show in different species (Witter et al., 2017). There is widespread agreement that the subiculum is the output gateway of the hippocampus. From there, neuronal activity is fed back to the entorhinal cortex, but many fibers leaving the hippocampal formation converge to the fornix and reach the anterior olfactory nuclei, the septal region, rostral portions of the hypothalamus, and fan out to the VMH to reach eventually the mammillary bodies and anterior (limbic) nuclei of the thalamus (Poletti and Creswell, 1977; Saunders and Aggleton, 2007). The trajectory of the postcommissural fornix is paralleled by the stria terminals pathway from the amygdala. Thus, the basal forebrain and hypothalamus are targeted in mammals by many efferent fibers from both hippocampus and amygdala, thought to exert downstream control over the motivational systems (Wang et al., 2021; Bang et al., 2022). This notion should be modified, however, as the septal nuclei and the hypothalamus contains many fibers that reach the hippocampus and limbic cortex parts upstreams (see Section A closer look at ascending hypothalamo–hippocampal connections).

#### Species differences in hippocampal inputs: Different neocortical partners

**Figure 4:**
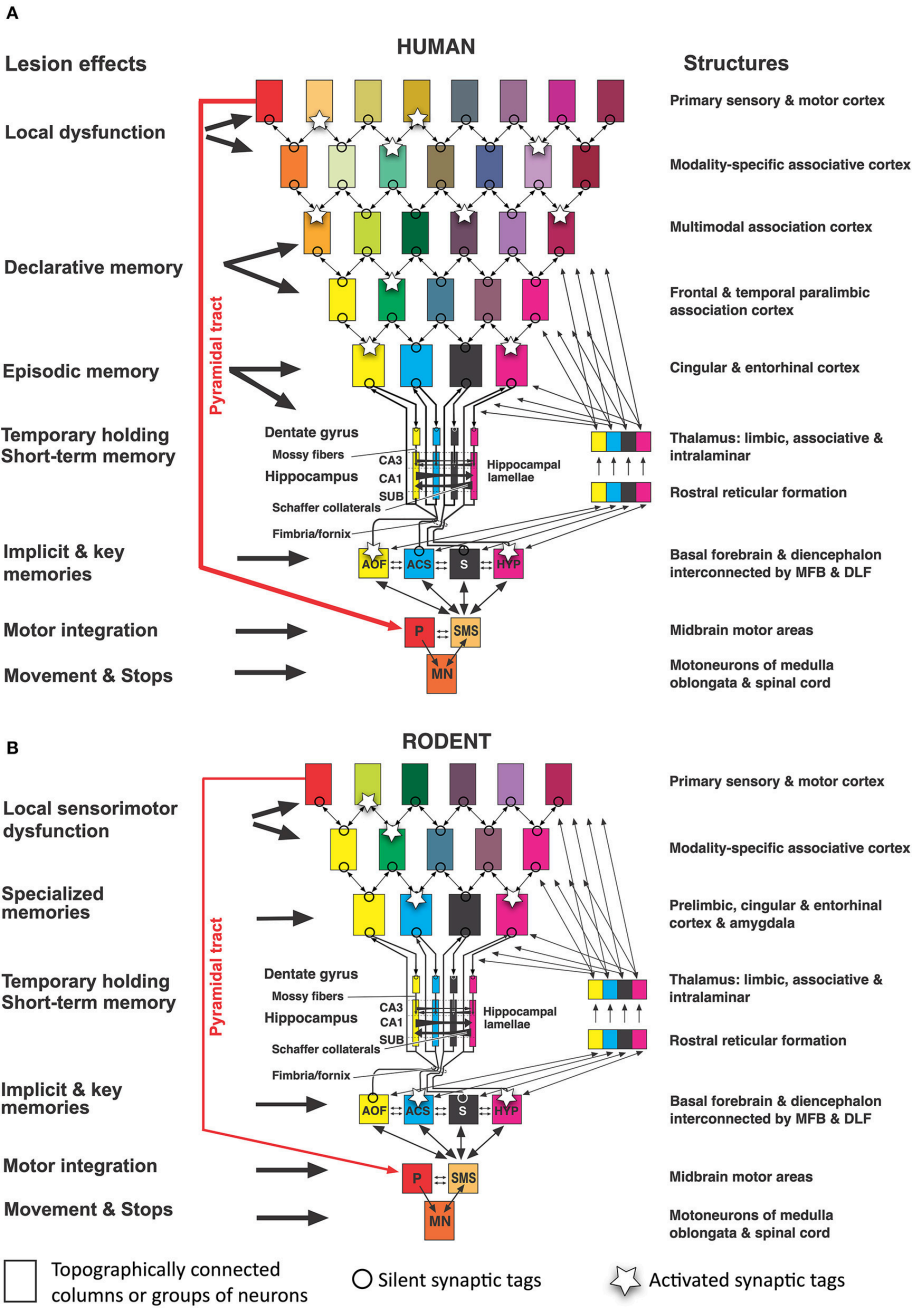
Converging information in the hippocampal formation of humans and rodents according to image reduction principles**. (A)** Human hippocampal loops receive reduced columnar activity patterns from cortex areas, aligning them to parallel (“lamellar”) loops that permit transformation of input/out patterns by Schaffer collaterals in CA3 and CA1. Different colors of rectangles indicate a progressive reduction of cortical activity pattern for representation in the hippocampal formation. Under idling cortical activity, the hippocampus transfers a copy of the activity pattern to basal forebrain and diencephalon that is fed back to the cortex. Under situations of alert, synaptic tags are activated and these tags help to form engrams or recalls (Lipp, 2015). **(B)** Corresponding view in rodents in which the input to the hippocampal loop system originates chiefly from non-associative cortex regions but also from subcortical structures. Thus, the hippocampal formation of rodents deals mostly with “non-cognitive” inputs from secondary sensory and motor cortex regions. Abbreviations: AOF, anterior olfactory nuclei; ACS, nucleus accumbens septi; CA1, hippocampal subfield; CA3, hippocampal subfield; HYP, hypothalamus, MFB, medial forebrain bundle; DLF, dorsal longitudinal fasciculus; MN, motoneurons; P, Pyramidal motor system; S, septal nuclei; SUB, subiculum; SMS, supraspinal (mesencephalic) motor nuclei. Parts of this figure have been published earlier in a Frontiers journal (Lipp, 2017) but this version contains new features.

When comparing mice and humans, it is often overlooked that their neocortical input patterns to the hippocampal formation are different, while the connections between hypothalamus and hippocampus appear to be largely similar. Figure 4 shows a simplistic concept of information processing from neocortex to hippocampus. The salient feature is that the widespread activity patterns of the cortical columns must be reduced when approaching the entorhinal cortex by a process resembling digital JPG image reduction (Mulligan, 1997), from where they are fed into a stack of lamellar loops. This reduction process is governed by existing or newly formed synaptic tags (Frey and Morris, 1997; Redondo and Morris, 2011). In this view, the hippocampus receives a much-reduced copy of the neocortical activity pattern which can be fed back to the cortex but is also communicated to the basal forebrain and hypothalamus. Depending on the signals evoking tag activity, primitive memories can thus be stored even at the hypothalamic level (Krzywkowski et al., 2020). The concept is debatable, but it is obvious that the rodent hippocampus receives information that is mostly derived from the rather small associative cortex regions dealing with sensation and movements yet not cognition. In contrast, the human hippocampus receives inputs that were preprocessed through complex multimodal association cortex in the frontal, parietal, and temporal lobes, and is thus getting “cognitive” input. Yet, this view also implies that the observable output of “hippocampal” tests in rodents may not show the cognitive causation expected by the translational scientist.

#### A neglected feature for translational research: Mossy fiber distribution and behavior

The trait showing maximal species differences and being thus of potential importance for translational research is the mossy fiber distribution within CA3. In fact, individual and genetic variation of the mossy fiber projection originating from dentate granule cells correlates with a variety of rodent behaviors, specifically the extent of the intra/infrapyramidal projection (IIP-MF), synapsing on basal dendrites or pyramidal cell bodies in the CA3 region (Figure 3D), for reviews see the study by Lipp et al. (1989) and Lipp and Wolfer (1999). In brief, variations of IIP-MF responded to selective breeding for two-way avoidance and remained correlated with this behavior in various studies including mouse strains, individual mice, and after postnatal manipulation. Likewise, genetic studies showed that mice with small IIP-MF projections were exploring less (Crusio et al., 1989) and correlations with radial maze learning were frequent (Schwegler et al., 1991; Crusio and Schwegler, 2005). Later studies revealed correlations with other tasks sensitive to hippocampal lesions, such as water maze learning (Schöpke et al., 1991), but variations of the IIP-MF were also found to correlate with different non-cognitive tasks including strength and asymmetry of paw preference (Gruber et al., 1991) and latency to attack intruders (Guillot et al., 1994; Sluyter et al., 1994). These findings did not easily fit into cognitive theories of hippocampal function. Qualitatively, it appeared to us that the extent of the IIP-MF was correlated with predictability of ongoing behavior: mice with larger IIP-MF were less distracted by noises and external stimuli and thus more successful in rather complex tasks, while individuals or strains with small IIP-MF appeared to react stronger when facing distracting or fear-inducing stimuli. Nonetheless, as argued by Crusio and Schwegler (2005), the multitude of observed correlations strongly suggests a causal physiological effect of mossy fiber variations on behavior.

**Figure 5:**
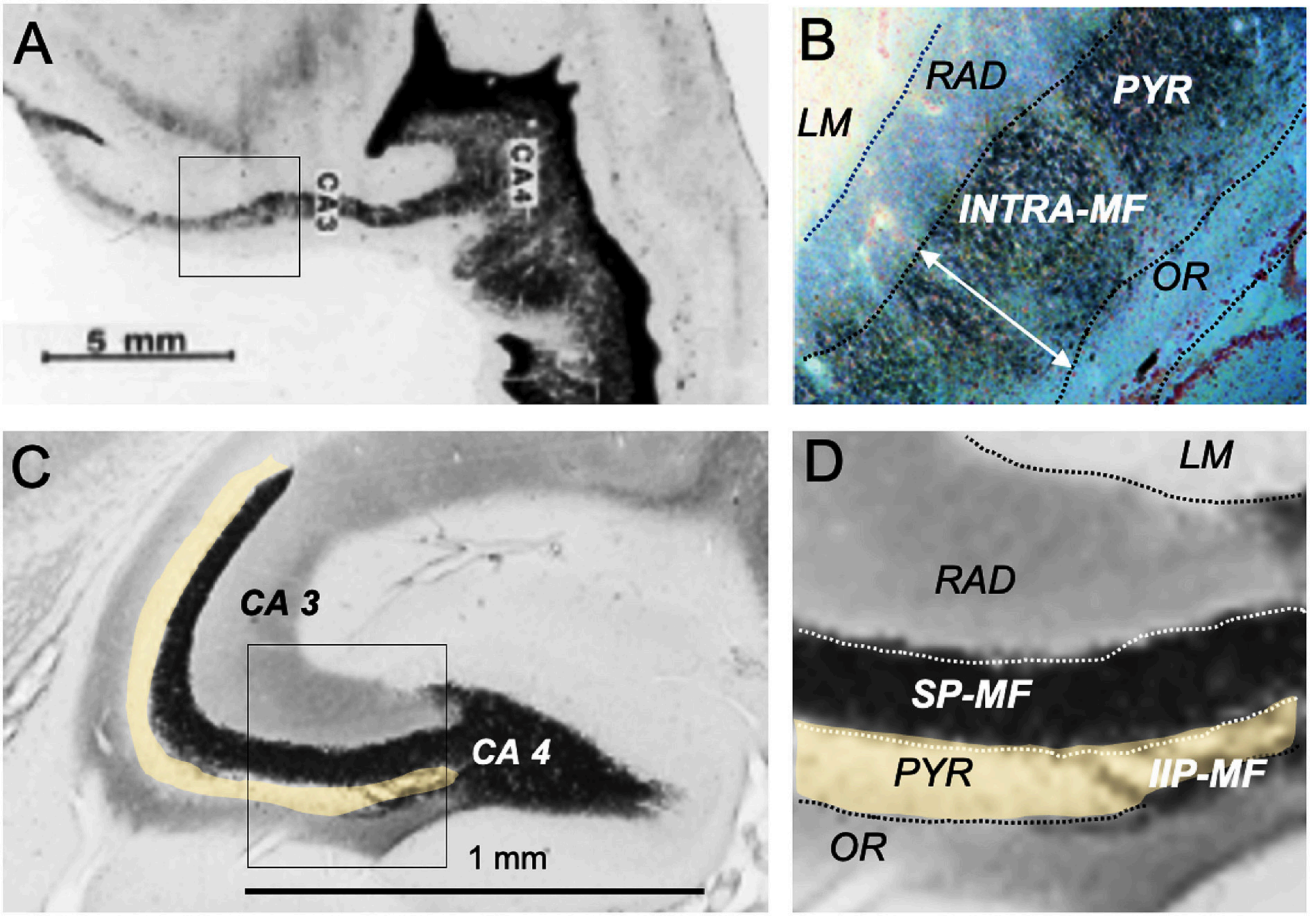
Hippocampal mossy fiber distribution in the human and the mouse brain. **(A)** Postmortem Timm staining of a longitudinally sectioned human hippocampus. Own unpublished data. **(B)** Inlet from **(A)** shows a continuous mossy fiber band pervading the entire pyramidal layer (neutral red counterstain). **(C)** Horizontal Timm-stained section from the mid-septotemporal layer of a house mouse. The unstained pyramidal cell layer is marked in yellow. **(D)** Inlet from **(C)** shows a clear separation of the suprapyramidal mossy fiber layer (SP-MF) from the pyramidal cell layer (PYR), and a small and short intra/infrapyramidal (IIP-MF) mossy fiber bundle covering the basal dendrites. Note that the IIP-MF projection of rodents is larger septally and dwindles temporally. The main species difference is a much more massive human mossy fiber projection covering all neurons in the pyramidal cell layer of CA3. INTRA-MF, intrapyramidal mossy fibers; LM, stratum lacunosum-moleculare; RAD, stratum radiatum; OR, stratum oriens.

By (non-systematically) inspecting many species, we observed enormous variation of mossy fiber projections across species (Supplementary Figure S2) suggesting that the mossy fiber input was larger in predatory or encephalized species, appearing mostly dependent on the size of the dentate gyrus. Figure 5A compares the mossy fiber patterns in a human hippocampus with that of a mouse taken at a mid-septotemporal level (Figure 5C). In the human hippocampus, the mossy fibers pervade the pyramidal cell layer from the beginning till the end of CA3 (Figure 5B, while the pyramidal cells in mouse CA3 are only partially covered by mossy fiber boutons (Figure 5D). Given that simple measures of the IIP-MF projection had often correlated well with many behaviors of mice, one might infer that the massive mossy fiber input in the human CA3 region might reflect a much stronger control of the pyramidal cells by mossy fiber input. But explanations must remain speculative, while a translational approach might be feasible (see Section The hippocampus as a sensor of malaise and well-being).

#### Experimental natural selection of mossy fiber distributions and associate behaviors

**Figure 6:**
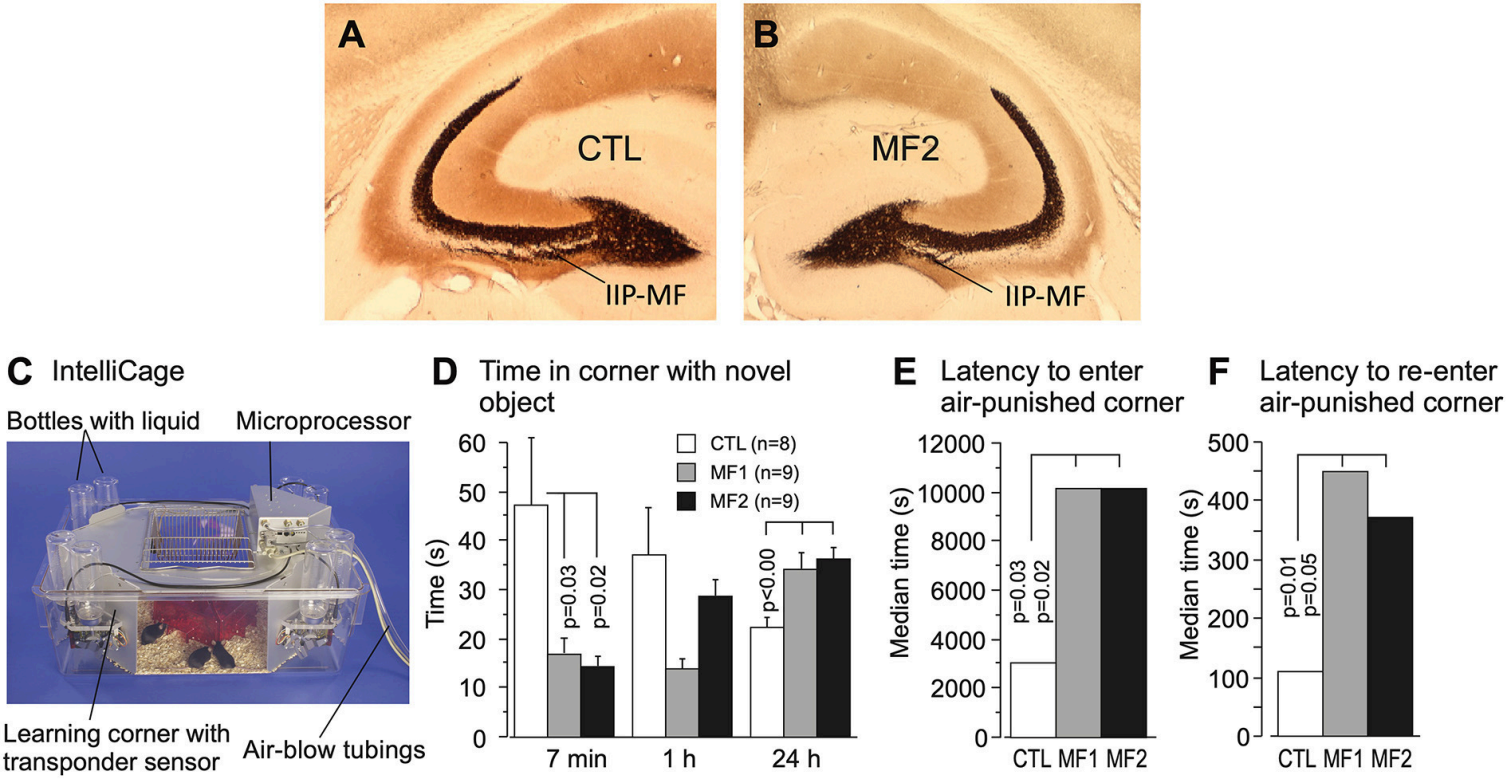
Natural selection of the hippocampal mossy fiber distribution in laboratory mice exposed to semi-naturalistic environments in Russia (Lipp and Wolfer, 2013) and behavioral changes in later generations raised in the laboratory and tested in a home-cage system. **(A)** Mice raised in parallel to the feralized animals but living in an animal house showed relatively large intra/infrapyramidal mossy fiber projections (IIP-MF) synapsing on basal dendrites. For details see Figure 5. **(B)** The feralized mice showed a significantly reduced IIP-MF projection and reduced exploratory activities in standard tests that persisted after embryo transfer in Switzerland. **(C)** Automated homecage system (IntelliCage^TM^) used for testing non-handled mice living in social groups (Kiryk et al., 2020). Mice carry transponders recording visits and water consumption in corners permitting operant conditioning but also assessing spontaneous reactions. **(D)** For this test, a novel object (a nut) was placed in one of the familiar corners for measuring the novelty reaction. Note that during the first 7 min the descendants of the naturally selected mice did not enter that corner much, while the control lab mice appeared very interested but lost interest thereafter, while the MF1 and MF2 were inspecting that corner more after 24 h. **(E)** The experiment started with randomly released and noisy air puffs in the corners, disturbing the mice. Control animals took about 1 hour to enter any one of the corners after which they received an air puff, while the MF1 and MF2 mice took many hours. **(F)** The CTL mice re-entered the corner in which they had received punishment significantly faster than the MF1 and MF 2. Except for some preliminary data, most of the results have not been published. The IntelliCage experiment was designed and conducted by Ewelyna Knapska, Warsaw.

The long series of mossy fiber studies abutting into more and more apparently mossy fiber-dependent behaviors led to the question whether variations of this trait were truly functional or became just visible by cherry-picking of standard behavioral tests. Therefore, four mouse strains with large or small IIP-MF projections were crossed systematically to obtain a founder population with equal proportions of genetic variance (a so-called diallel cross) and the F1-animals were transported to a Russian field station and released in two outdoor pens (Lipp and Wolfer, 1999, 2013) while a control population obtained by interbreeding the same founders was kept in a standard mouse facility. The results were instructive (Figure 6). After 2 years (5–6 generations of outdoor life), the mice living in the outdoor pens had developed significantly reduced IIP-MF projection and this persisted up to the fourth year. The shift in IIP-MF was not due to environmental factors, because mice from the three groups underwent embryo transfer and were maintained as ordinary mouse lines in the same animal facility in Switzerland. They also underwent a series of behavioral tests. One of them conducted in an automated home-cage testing system (Kiryk et al., 2020) proved instructive because the mice were tested without handling, and members of all three lines were together in an IntelliCage (Figure 6C). This study revealed that the naturally selected mice were reluctant in approaching a harmless novel object (a nut) placed in one of the corners while the non-selected mice approached it frequently but lost interest rapidly. However, after 24 h, the naturally selected mice were visiting the object more frequently, implying that natural selection had shifted a genetically dependent balance in exploratory tendencies, perhaps by altering a hypothalamic set-point (Figure 6D). A further test assessed the behavior of the mouse lines after the introduction of randomly occurring noise in the corners (generated by air-puffs), and delivery of an air-puff when a mouse had finally entered a corner. All mice hesitated before visiting a corner, but the naturally selected lines took 5 h more (Figure 6E). Re-entering a punished corner was dramatically delayed in these mice as well (Figure 6F). Thus, smaller IIP-MF on basal dendrites remained correlated with an altered response pattern in animals facing threatening or stressing situations as it was predicted by the very first studies focusing on IIP-MF variation and exploratory behavior (Crusio et al., 1989). It can be added here that small IIP-MF projections were most often correlated with superior two-way avoidance learning but poor performance in radial maze learning (Crusio and Schwegler, 2005), while apparently superior cognitive abilities of mice with larger IIP-MF projections, as judged by observing them in radial and water mazes, were less useful for survival under real-life conditions. Whatever the interpretation, the study proved that natural selection could act surprisingly fast in changing hippocampal circuitry and behavior, and that variation of the IIP-MF appeared to be truly associated with meaningful variations of mouse behavior outdoors and indoors.

## Anthropomorphizing mice entails logical fallacies and wrong conclusions

**Figure 7:**
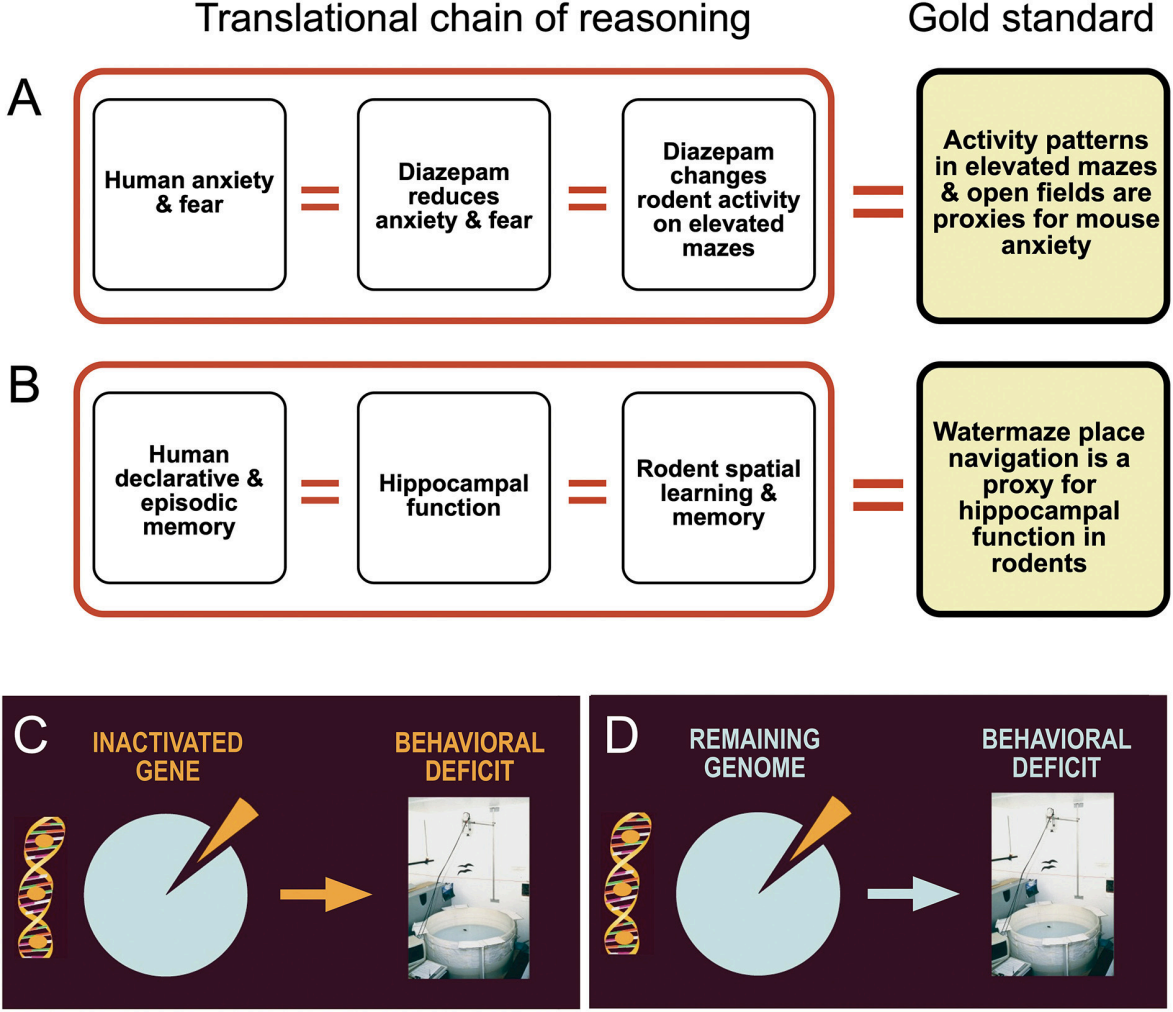
Logical flaws and inadequate assumptions in translational behavioral brain research. **(A)** Drugs that lower human anxiety and fear are given to animals. If these drugs alter motor activity in tests assumed to measure anxiety, scores are taken as a proxy for anxiety levels. **(B)** Human declarative and episodic memory is thought to depend on proper hippocampal function. Since hippocampal lesions in rodents cause impaired spatial learning, variation in water maze place navigation is used as a proxy for hippocampal memory functions. **(C)** The mechanistic standard assumption is that a removed or inactivated part of the brain is responsible for the control of specific behaviors. **(D)** The real situation shows that the behavioral change is a joint function of the remaining genome or brain circuitry. Many processes underpinning a behavioral change after invasive manipulations are unknown and thus much of the observed changes could be caused partially by unpredictable chance events.

A major cognitive problem pervading translational approaches is illogical shortcuts, shown graphically in Figures 7A,B. The main point is that functional deficits in motivational systems or cognitive processing as observed in humans are extrapolated to mice. The social part of the problem is that behavioral testing in a translational context is often done collaboratively, specifically for genetically modified mice. Molecular biologists wishing to have their mice tested for translational potential are often dissatisfied with complex explanations of behavior. Tests such as fear conditioning or water maze learning offer much face validity because most lay people quickly grasp that a probe test in the water maze proves the existence of spatial memory or freezing in a chamber of previous shock experience a memory engram. However, they are mostly unaware that these measures are generally quantitative and verified by statistics only. But memory is not easily quantified in behavioral tests and performance variation supposed to reflect memory or lack thereof is often based on coping strategies or species-specific priorities of situation-dependent action as explained below.

### Ethological priorities of mice: Stop or go!

**Figure 8:**
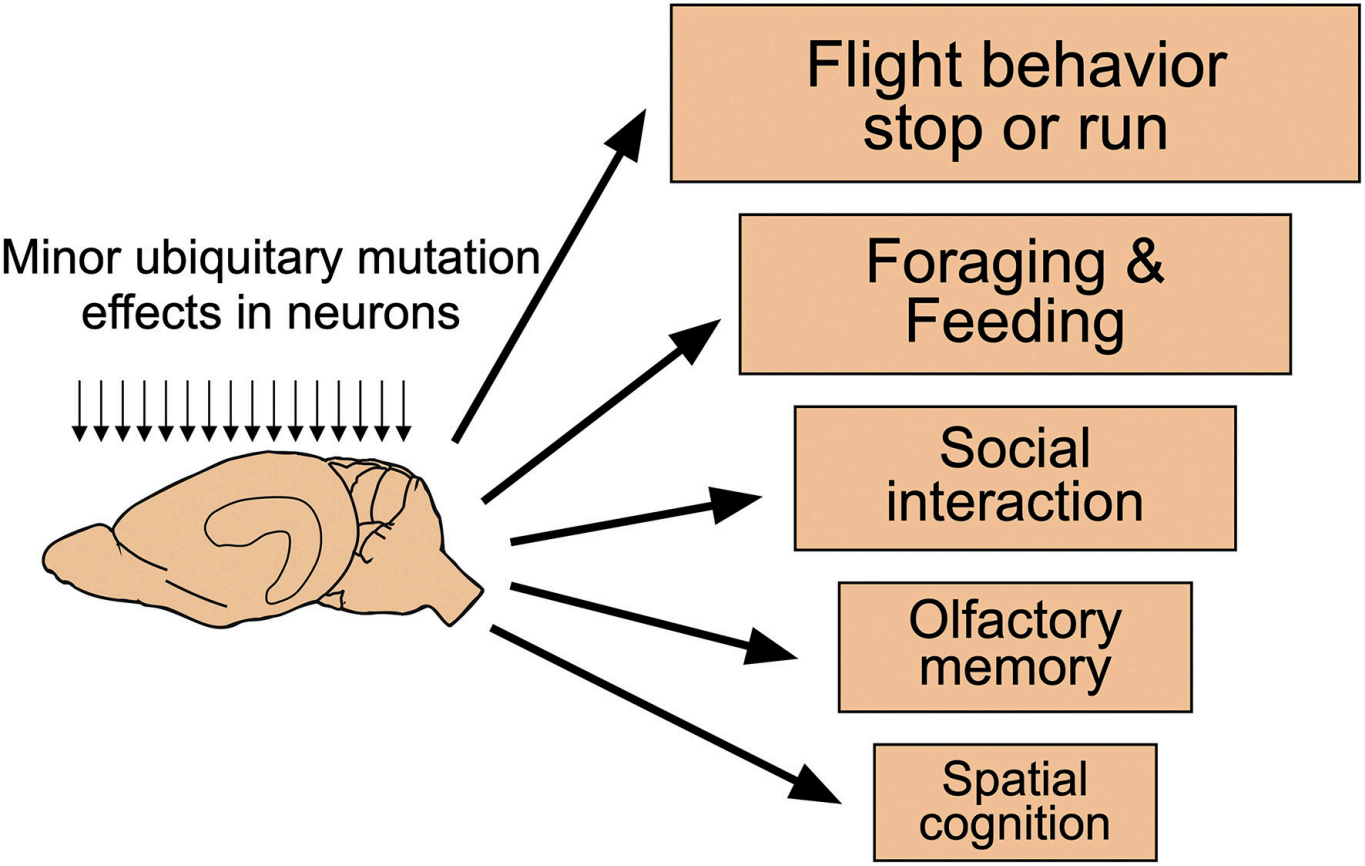
Functional hierarchical levels in the daily life of mice. Many targeted mutations affecting neuronal functions across the entire mouse brain are likely to show phenotypic changes according to an (arbitrary) functional hierarchy of behavioral changes. Most important for mice is the regulation of freezing or running which is governed by many brain systems. Thus, even subtle changes at the cellular level are likely to be observed but their causation remains unclear. On the contrary, changes in spatial memory and cognition are unlikely to show up in such spontaneous behavior. Modified from Lipp and Wolfer (2013).

In classifying behavior of mice and men, it is often useful to distinguish between behavioral traits used in daily or monthly life from these important during a lifetime or evolution. Figure 8 shows a somewhat arbitrary hierarchy of behavioral processes potentially influenced by rather minor experimental interference, such as drugs or many targeted mutations. Most important for the daily life of mice is a very rapid decision to freeze or rush when dealing with predators or aggressive conspecifics (Kondrakiewicz et al., 2019). Large animals and humans have the freedom of mulling when facing conflicting cues and they may choose to remain passive or active, revising the decision if required. As mice face rapid predators or conspecifics, they are likely to enter immediately a state of immobility or rushing. Humans are inclined to believe that such behavior is caused by or at least associated with an emotion, chiefly fear and anxiety. But it could also happen reflexively without emotions that may form later. This ethological antagonism facilitates the detection of comparatively minor experimental effects in many brain systems not necessarily related to memory or fear. It seems reasonable to assume that these antagonistic innate behavioral responses are under some form of inhibitory control, probably by tonically active GABA-ergic axons from the reticular part of the substantia nigra. A sudden rush is easier initiated by disinhibition of structures such as the mesencephalic locomotor region (Esposito and Arber, 2016) than by building up increasing pressure to move. Likewise, behavioral arrest can be produced rapidly by GABAergic systems. This constellation may hinder translational research, specifically testing of drugs aimed at reducing anxiety and improving mood. These may result in lowering anxiety of mice, but if they interfere with GABAergic mechanisms, some results may be unexpected. In humans, paradoxical effects of diazepam such as hyperactivity, hostility, and aggression have been observed, but the incidence appears to be low (Paton, 2002). The situation may be different in mice. For example, keeping adult male mice isolated for more than 4 weeks alters the subunit expression of GABA type A receptors (GABAA-R) and increases their locomotor activity following exposure to diazepam or imidazenil (Pinna et al., 2006). Since the pharmaceutic industry has a preference to work with male mice and these often need separate housing, other types of drugs supposed to act *via* GABA receptors may elicit no or paradoxical responses as well. This might at least partially explain why 17 out of 19 mouse tests designed to measure anxiolytic effects were found to be highly unreliable (Rosso et al., 2021).

### Ethological priorities of mice: Moving alone or together?

The second-most important behavior in the daily life of a mouse is foraging and feeding, driven by energetic needs. However, it also means that mice have a strong drive for moving. This may conflict with measures of memory assessing the duration of freezing after shock-exposure: the assumption that mice that start moving in the shock-box have a poor memory might simply reflect a stronger intention to get out of it. But there is little doubt that mice like to move. When given a running wheel, they may run some kilometers per day, although there are large interindividual differences even among inbred mice. But their penchant for moving poses a problem in rewarded learning tasks (e.g., radial mazes) as they seem not much concerned by moving as long as there is a partial reward. They also tend to use win-stay strategies by visiting repeatedly arms with consumed bait, while rats, crows, and juvenile rabbits rely on win-shift strategies (never visit a baited arm again) as shown by a study using a giant outdoor radial maze (Lipp et al., 2001). Unsurprisingly, hedgehogs and chickens, species relying on random walk strategies for foraging, were poor radial maze learners while guinea pigs remained immobile and could not be tested.

Social interactions during a day occur less frequently. Rodents often huddle together to save energy or to maintain social contact. A less known feature is that foraging in mice often occurs in social groups. Studies with transponder-tagged female mice in an outdoor pen with eight feeder boxes emulating a radial maze situation have shown that the animals established group territories, each group visiting only half of the boxes, thus thwarting the expectations of the experimenters (Dell'Omo et al., 2000).

While these three behavioral classes are dominating the daily life of mice, cognitive abilities such as spatial or olfactory memory probably play a minor role because the environment and smells of a mouse colony do not change quickly. Such abilities are doubtlessly important for long-term survival and reproduction, but in mice they have not be tuned for rapid adaptation and behavioral flexibility as required for predators.

## Bothersome unpredictable results in neurobehavioral brain research

Mechanistic thinking in the clockwork sense is still deeply rooted in brain research, specifically in molecular and genetic fields and synapse physiology. They are mostly appropriate there, but much less so in the domains of neuropsychology and behavioral brain research investigating behavior only. Leaving brain imaging and large-scale electrophysiological mapping aside, there seems to be a strong penchant to a mental short-cut in experimenters: they anticipate that their interaction with a given brain system results in a behavioral outcome related directly to the target structure (Figure 7C). One can suspect that most observers know that the observed behavioral reaction is a response either from the remaining brain, remaining genome, or being caused by compensatory homeostatic processes (Figure 7D) but that knowledge is rarely communicated. Part of it is simply peer pressure arising from cooperating molecular biologists or clinicians: they do not like to learn that their efforts might be marred or blurred by unpredictable processes. The usual excuse of the behavioral specialist is then mostly statistical. There must be a specific mechanistic effect but the tools for measuring are not good enough so that unpleasant variability is evident. Essentially, this is an engineer's view in which the terms standard deviation and standard error reflect imprecision of measurement. But the possibility of truly stochastic (statistically unpredictable) processes masking phenotypes after whatever treatment should not be ruled out. Therefore, this section deals with one example of unpredictable events following gene deletions.

**Figure 9:**
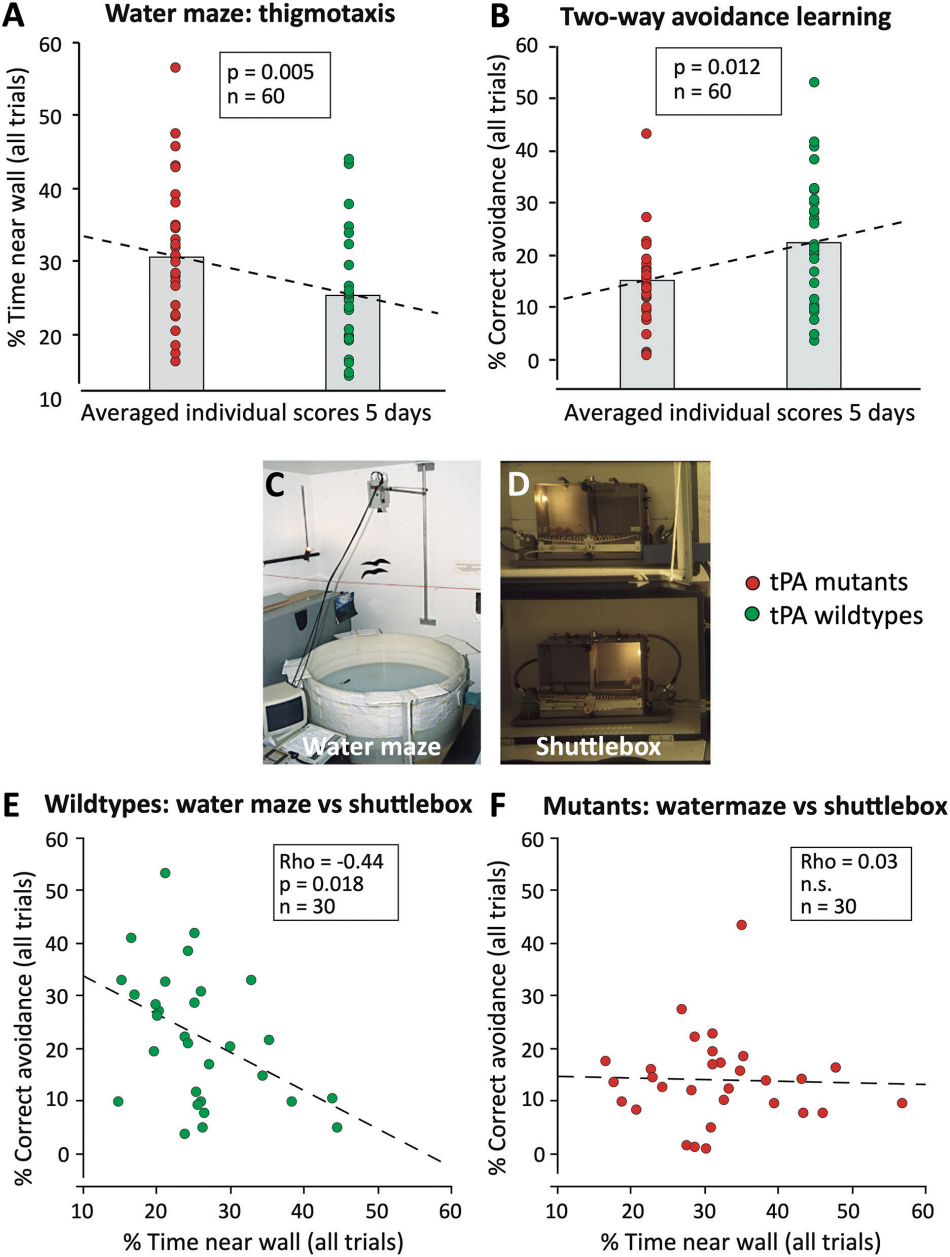
Spurious parallel behavioral effects in conventional tissue-plasminogen knockout mice (tPA KO) that were observed in two hippocampus-dependent tasks (Huang et al., 1996). **(A)** tPA KO mice showed significantly increased wall-hugging (thigmotaxis), a behavior that impairs water maze learning. The data were plotted individually, the column refers to the usual bar plots showing the mean of a score. Note that about half of the mutants showed equal scores as the wild types. **(B)** Subsequent testing of the same mice in another hippocampus-dependent task (two-way avoidance or shuttlebox learning) showed parallel effects of the mutation. Since two-way avoidance is usually increased after hippocampal lesions, the individual scores were tested for correlations assuming that the parallel differences should reflect a common deficit due to hippocampal problems. **(C)** View of watermaze. **(D)** View of shuttle boxes wherein mice must change to the other side after illumination of the compartment, the light always going on where the animal is. **(E)** Wildtype mice showed a reasonably strong correlation between reduced thigmotaxis and better two-way avoidance learning, suggesting a physiological relationship between the processes mediating the two tasks. **(F)** In mutant mice, the correlation was lacking, indicating that a score observed in one test does not predict scores in another test. The difference between the scatterplots suggests that in mutants compensatory yet unpredictable processes during brain development were accounting for some of the behavioral differences. Original data were published (Huang et al., 1996), but not the presented re-analysis of the individual scores.

Figure 9 shows a re-analysis of earlier data obtained by characterizing electrophysiologically and behaviorally mice lacking the gene for tissue plasminogen activator, tPA (Huang et al., 1996). The mice had been tested by us in a standard water maze procedure, and the same animals subsequently for two-way avoidance (shuttlebox) learning (Figures 9A–D), while long-term potentiation (LTP) in hippocampal slices was investigated in the United States. Given that, we observed significant group differences in both tests, we had assumed that the tPA deficiency was affecting a common underlying factor, possibly LTP, accounting for the behavioral phenotypes. However, when presenting the results as individual data scores (Figures 9A,B) we were asked why half of the knockout mice were showing similar scores as the controls and why the “outliers” were apparently responsible for a significant behavioral phenotype. Having no good answer, we then analyzed correlations between the individual data scores. If there would be a common underlying factor causing covariance of the scores in the two tests, the worst shuttlebox learners should also be the worst watermaze learners. This assumption held only for the controls that showed a moderate correlation between the scores (Figure 9E) but not at all for the knockout mice in which we found a zero correlation (Figure 9F). This implied that the tPA knockouts were compensating problems in the two tasks in different yet unpredictable ways and that the overlap between controls and knockouts showed that a substantial fraction of the tPA mutants were apparently able to compensate their deficit fully. Since then, all studies conducted by our lab always checked for such interactions.

However, the problem of spurious correlations between endophenotypes and behavior can be found in many studies claiming to find correlations without checking individual correlations. For example, there have been numerous studies linking experimentally manipulated rates of hippocampal neurogenesis with parallel behavioral changes, but out of some 900 studies, only eleven studies were measuring both the degree of adult neurogenesis and the behavioral scores in the same animals (Lazic et al., 2014). Thus, many claims of functional correlates between hippocampal neurogenesis and cognitive abilities might reflect spurious correlations (Lipp and Bonfanti, 2016).

## Back to hippocampus: Behavioral lesion effects not predicted by current theories of hippocampal function in mice

The following sections deal with the effects of complete bilateral and excitotoxic hippocampal lesions on mouse behavior. Obviously, complete removal of the hippocampus is a crude approach for assessing its role, but the seminal water maze experiment of Morris et al. (1982) was crucial in implementing the belief that also the rat hippocampus was necessary for cognitive performance, such as spatial mapping. Therefore, we initiated a series of studies to document the effects of complete hippocampal lesions in mice tested in the laboratory and under naturalistic conditions. For both sets of (largely unpublished) experiments, the hippocampi of mice were destroyed bilaterally by infusion of excitotoxic substances (Deacon et al., 2002), and mice with sham operations or removed hippocampi were observed over prolonged periods.

### Acute and chronic effects of hippocampal lesions in standardized water maze testing

**Figure 10:**
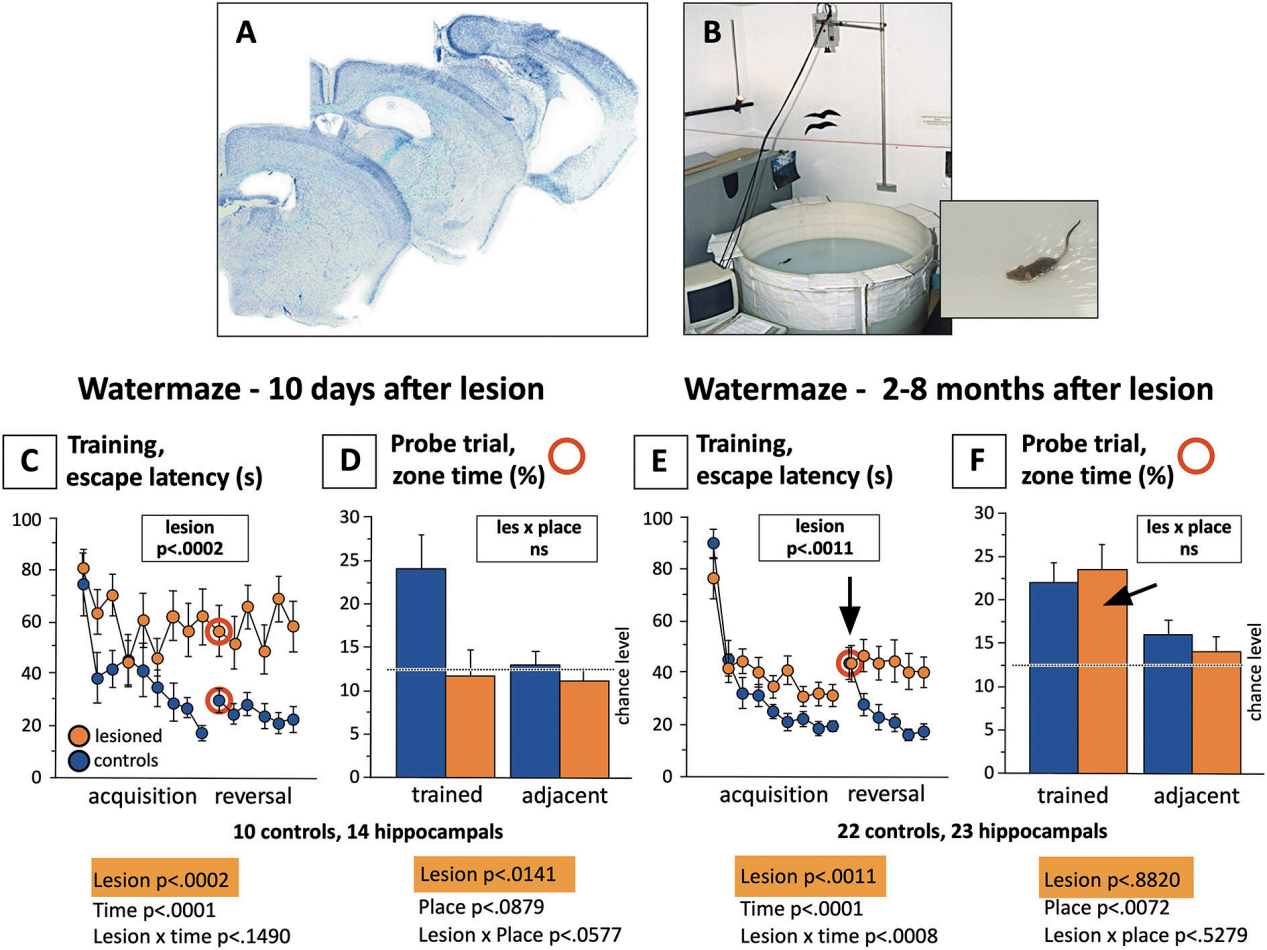
Water maze learning in fully hippocampectomized mice shortly after operation and in the chronic state. **(A)** Brain sections of mice in which the hippocampi were destroyed by bilateral infusion of excitotoxic substances. **(B)** Standardized water maze used for many years in Zürich to study thousands of mice. The procedure included 3 days with totally 18 trials to measure acquisition, followed by 2 days (12 trials) after reversal of the platform position. The first reversal trial was taken as a probe trial, analyzing the approaches to the former platform position (the classic proof of spatial memory), the rest of the reversal time served to assess the flexibility of the mice in adapting to a changed situation. The trial length was 120 s. Note that for better presentation, values for two subsequent trials were averaged, thus showing only 9 and 6 data points for acquisition and reversal. **(C)** Severe impairment (as expected) of lesioned mice to reach the platform, chiefly because of thigmotaxis (wall hugging). The reversal phase showed a clear memory effect in the controls followed by learning a new platform position. **(D)** Comparison of probe trial scores (red circled trials), showing random place preferences in the lesioned animals and significant differences to the controls. **(E)** After different recovery times, lesioned mice showed still significant differences in learning the task as compared to the controls (chiefly because of stronger thigmotaxis), but they show distinct learning curves, and a strong increase in search time after platform reversal. Thereafter, however, they appeared to be unable to orient toward the new platform position. **(F)** The “the gold standard” score for assessing spatial memory did not show any differences between the groups, both groups scoring significantly above chance levels. Unpublished data. Lesioning was performed in Zürich by Rob Deacon and Giovanni Colacicco.

Figure 10 summarizes these studies. The standardized water maze protocol included 3 days of learning to find a hidden submerged platform, each mouse having six trials of 120 s duration per day to assess improvement within and across days. At the beginning of the fourth day, the platform position was reversed, and this trial was taken as a probe trial for measuring approach preferences to the former platform position. However, the trials were continued for further 11 trials to assess navigational flexibility. Ten days after the operation, the controls showed rapid learning but the lesioned ones showed no acquisition, reflecting excessive thigmotaxis in an unknown environment. The probe trial showed clear spatial preference scores in the controls while the lesioned ones performed at chance levels. Various samples of mice including a total of 45 mice were tested 2–8 months after the lesion. There was again a significant lesion effect in learning: the controls were faster in learning, but now the lesioned mice also showed a clear learning curve, the difference due to stronger thigmotaxis (wall-hugging). Interestingly, the probe trial showed significant spatial preference scores in both groups, indicating that hippocampectomized mice could remember well the platform position but were unable to learn a new one. Thus, the long-term effect of removing large segments of the mouse brain was relatively subtle, a loss in behavioral flexibility in water maze learning. This insight was not new as it was shown that partial and selective recovery of navigational learning could occur after retrohippocampal lesions (Schenk and Morris, 1985), but this recovery was barely noticed by the neuroscience community. Likewise, studies showing that rats with complete hippocampal lesions could use alternative yet less-efficient systems for learning contextual fear (Wiltgen et al., 2006) did not overcome the dominant narrative of the hippocampus as the main substrate for memory in humans and animals.

### Hippocampal lesions, species-specific behavior, and circadian activities outdoors and in the laboratory: Some own data and explanatory hypotheses

#### Surprising findings

**Figure 11:**
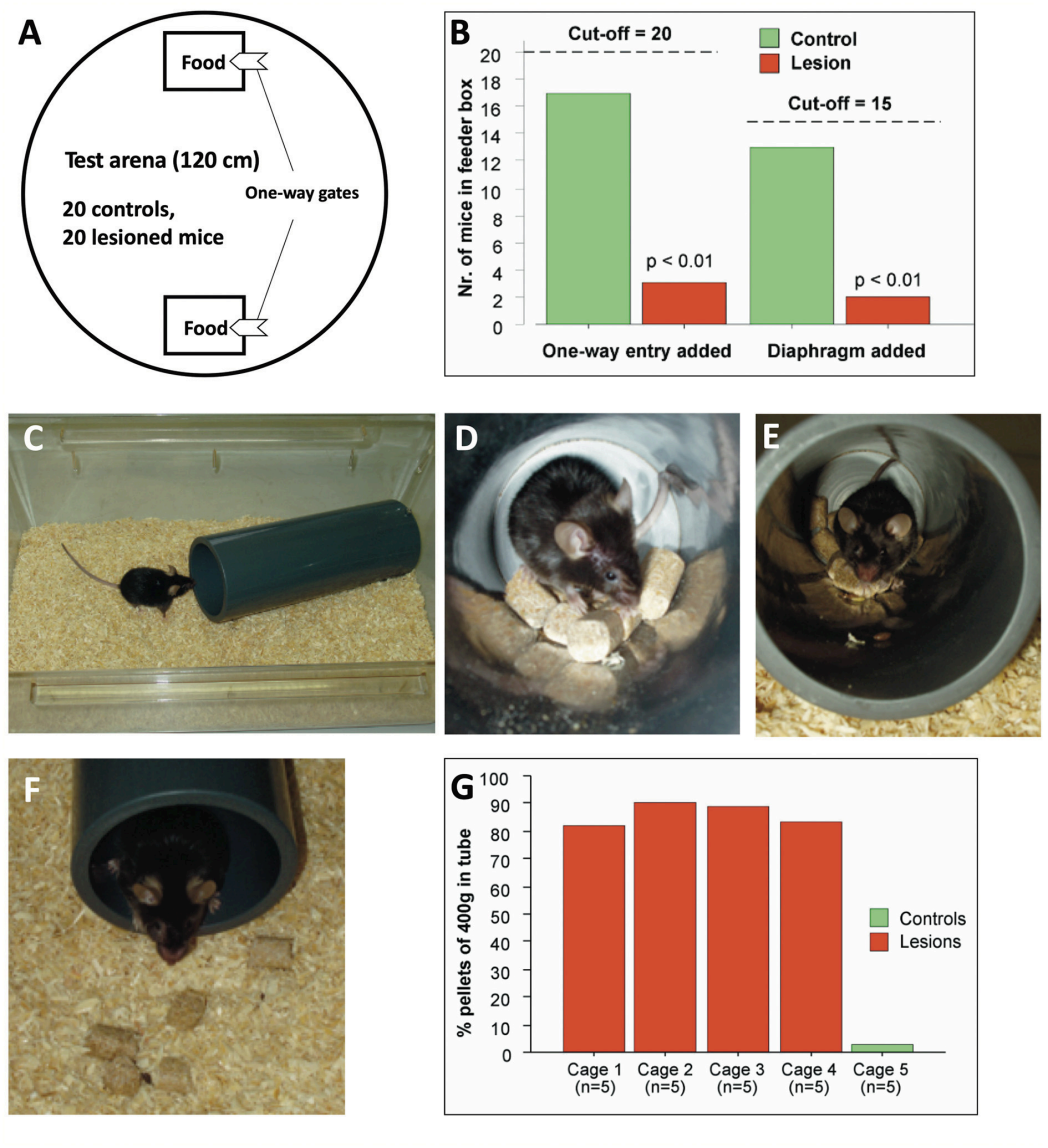
Behavioral testing of hippocampectomized mice in a Russian field station. Mice were bilaterally lesioned and underwent preliminary testing for spontaneous behaviors before placing them in an outdoor pen (see Figure 12) where they had to enter computer-controlled feeder boxes. **(A)** A first check was done by placing two familiar mouse cages equipped with one-way gates and containing food in a circular arena. The 40 operated female mice carrying transponders were placed at once in the arena, and the test was stopped when the observers counted 20 mice having entered test cages. The test was repeated 1 day later by adding a diaphragm to the one-way gate. **(B)** The first test revealed a highly significant difference in mice entering the cages, 17 controls, and only three hippocampally lesioned mice. After adding a diaphragm, the cut-off was set at 15 animals of which there were still only 2 lesioned animals in the cage. **(C)** The food-tube test for mice was designed by Deacon et al. (2002). A plastic tube was filled with 400 g mouse food pellets and placed overnight in a cage with one or several mice. Most laboratory mice begin an almost frantic removal of food pellets. **(D)** View of working mouse from inside. **(E)** Outside view with mouse carrying a pellet to the exit. **(F)** Dropping of the pellet outside. **(G)** Result of an overnight test with 25 mice. Because tubes were limited at the field station, each cage contained five mice. The (sham-operated) control mice practically cleared the tube, whereas among the lesioned ones, only a few mice removed some pellets. Note that they were physically not handicapped and active. Mice were operated by Rob Deacon, tested for adaptation by Mike Galsworthy, and pictures of the food-tube test were made by Giovanni Colacicco.

Inspired by the development of tests assessing species-specific behavior following hippocampal lesions in mice (Deacon et al., 2002; Deacon and Rawlins, 2005), outdoor studies in a Russian field station were initiated to assess known and unknown lesion effects. Most of the data are not published yet. In one study, after about 20 days of postlesional recovery time, 40 female mice (20 hippocampal, 20 sham controls) were shipped to Russia and brought to the field station. They tolerated the trip surprisingly well and underwent adaptation procedures to one-way gates enabling them to pass later through gates leading into computer-controlled feeder boxes placed in outdoor pens. They were all placed together in a circular arena that contained two mouse cages with attached one-way gates (Figure 11A). The cages were removed after 20 mice had entered them, and the group identity was verified by transponder reading. Figure 11B shows the results of this superbly fast collective test of hippocampal malfunction: among the first batch entering the cages, there were only three with hippocampal lesions. The gates were then modified by adding a plastic diaphragm. Because it took more time for all mice to enter the cages, the cut-off point was set to 15 mice, out of which there were only two lesioned mice. Overnight testing of mice using the Deacons pellet removal test (Figures 11C–F) confirmed the sensitivity of this simple test in revealing hippocampal lesions (Figure 11G).

**Figure 12:**
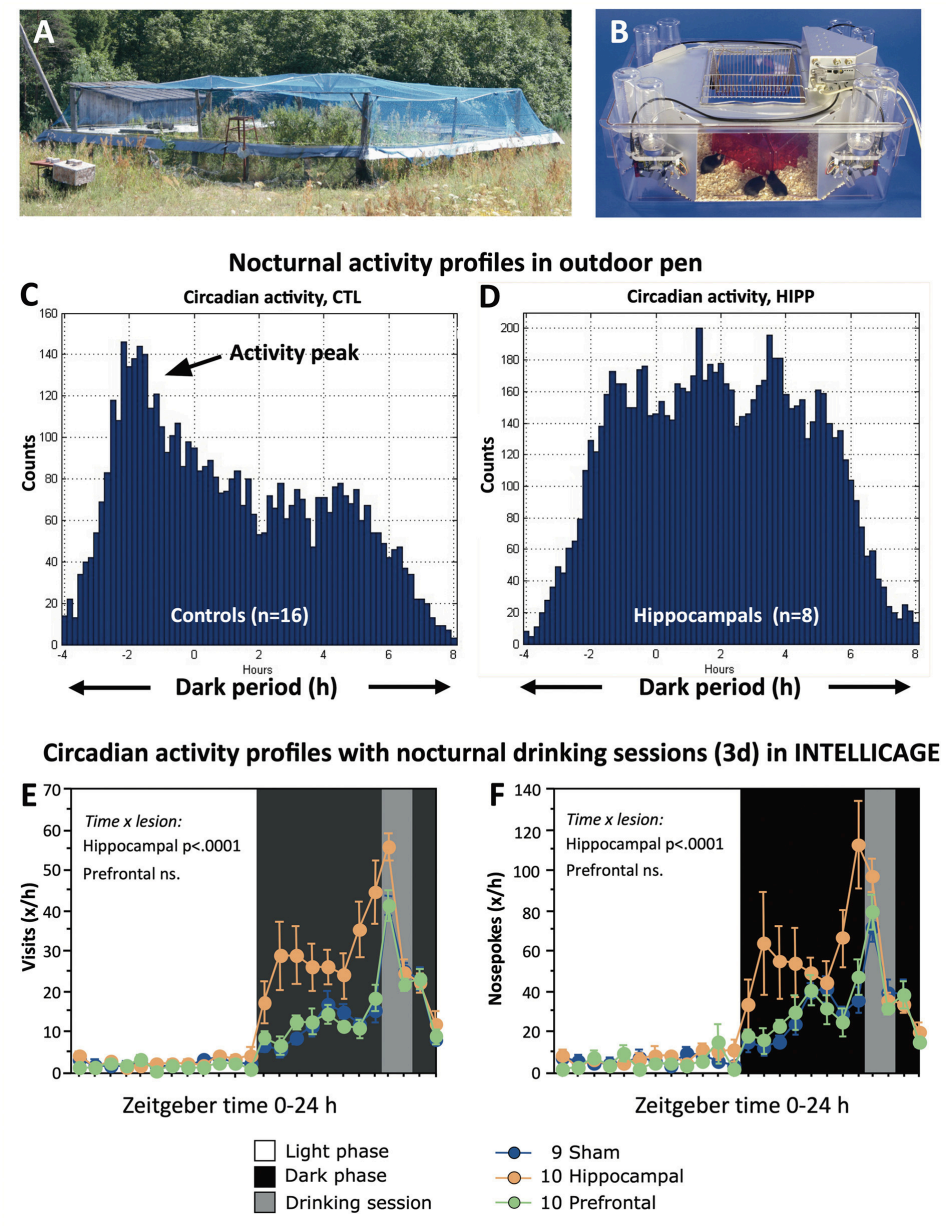
Impaired regulation of circadian activity of hippocampectomized mice in seminaturalistic and laboratory environments. **(A)** Small outdoor pen in Russian field station for studying hippocampectomized mice (see also Figure 11). The pen was covered by a net to block avian predators. For further details, in an experiment using genetically modified mice in the same set-up, see also the study by Vyssotski et al. (2002). **(B)** Automated homecage system (IntelliCage^TM^) routinely assesses circadian activity by corner visits and drinking (Kiryk et al., 2020). Normally, water is available *ad libitum*, but here access to water was allowed for 1 h in the night only to check how experimental animals would tune their activity patterns to specified time windows. **(C)** Nocturnal activity of sham-operated as counted by visits of transponder-tagged mice in eight placed feeder boxes. Note a strong initial peak after darkness followed by a gradual decline. **(D)** Hippocampally lesioned mice showed a strong increase at the beginning of darkness. Afterward the mice were highly active during the entire night. Note that the Y-axes are not scaled equally. **(E**,**F)** Circadian activity profiles of controls and mice with either prefrontal or hippocampal lesions (chronic stage). As in the outdoor pens, the hippocampus-lesioned mice started with high activity by visiting corners and nosepoking there and increased their activity gradually toward the time window for drinking. In contrast, both controls and prefrontals appeared to have a much more precise timing by increasing their activity just a short time before opening the drinking session. Experiments in Russia were done by Alexey Vyssotski, Mike Galsworthy, and late Nada Ben Abdallah. For the Experiments with IntelliCage see (Voikar et al., 2018).

Despite the precaution in pre-adapting the mice, about half of the lesioned ones died within 1–2 days after transfer to the pen (Figure 12A) for unknown reasons. The others adapted and the losses of them or of the control mice remained minimal over a period of 45 days. Taken together, the behavior of the lesioned mice was characterized by poorly structured nocturnal activity and by repeated visiting of the same box even though they would no longer receive food. Figure 12C shows an observed nocturnal activity pattern of mice in an outdoor pen: a marked activity peak at the begin of darkness, and a less-pronounced second peak in the mid of the night. In contrast, the hippocampally lesioned mice became very active soon and maintained a high activity level throughout the night (Figure 12D). The observed deficit in activity timing was analyzed later in more detail in the laboratory by using IntelliCages^®^ housing female mice with sham lesions, prefrontal, and hippocampal lesions together. Their lesions were chronic, and they lived in these home cages for prolonged periods (Voikar et al., 2018). To check their timing abilities, they had an external zeitgeber by delivering water during corner visits for 1 h in the night only. Figures 12E,F shows that the controls and the prefrontally lesioned mice kept relatively low activity at the begin of the night but increased it by visiting corners and nose-poking for water specifically in the hour before water delivery. In contrast, the hippocampus-lesioned mice started with high activity levels that became reinforced before drinking time (probably because of the behavior of their cage mates). Thereafter, all mice became rapidly inactive.

#### How to interpret such observations?

One factor is the huge behavioral variability observed in the hippocampally lesioned mice, specifically in complex situations, such as water maze and IntelliCage^®^. Partially, this may reflect incomplete lesions as there was no possibility to check the lesion size in the field station. However, most lesions were done by experienced persons, so this factor might not be decisive. Rather, it appears that the variation in many behavioral scores reflects a genuine inability to stabilize behavior in complex situations, so that a variability index as used in stock markets might be a better descriptor. On the other hand, the predictability of behavioral changes following lesions in seemingly non-cognitive species-typical behaviors is astounding and may indicate the involvement of hippocampus-dependent processes evading explanations in terms of experimental neuropsychology. For example, the unwillingness of lesioned mice to enter a one-way gate may reflect vibrissal hypersensitivity, a fear reaction, or something else. In any case, it seems to be a reliable and predictable trait that has been described in detail (Deacon and Rawlins, 2005). It appears that passing narrow doors may have a significance for mice that eludes the human observers.

Nobody knows what drives a normal C57BL/6 mouse to clean frantically a tube from food pellets or other kinds of pebbles, while a lesioned mouse of the same strain is not handicapped in sensorimotor abilities but unresponsive to the situation. Possibly, such hippocampal deficits in natural behaviors reflect a broken interaction between an ethological releasing stimulus and a corresponding species-specific behavioral pattern. But this (old-fashioned) view would imply that the interplay of the hippocampus and the basal forebrain structures (inclusive hypothalamus) had evolved under the demands of the basal forebrain to have computational extensions.

### Primordial functions of the hypothalamo–hippocampal loops?

**Figure 13:**
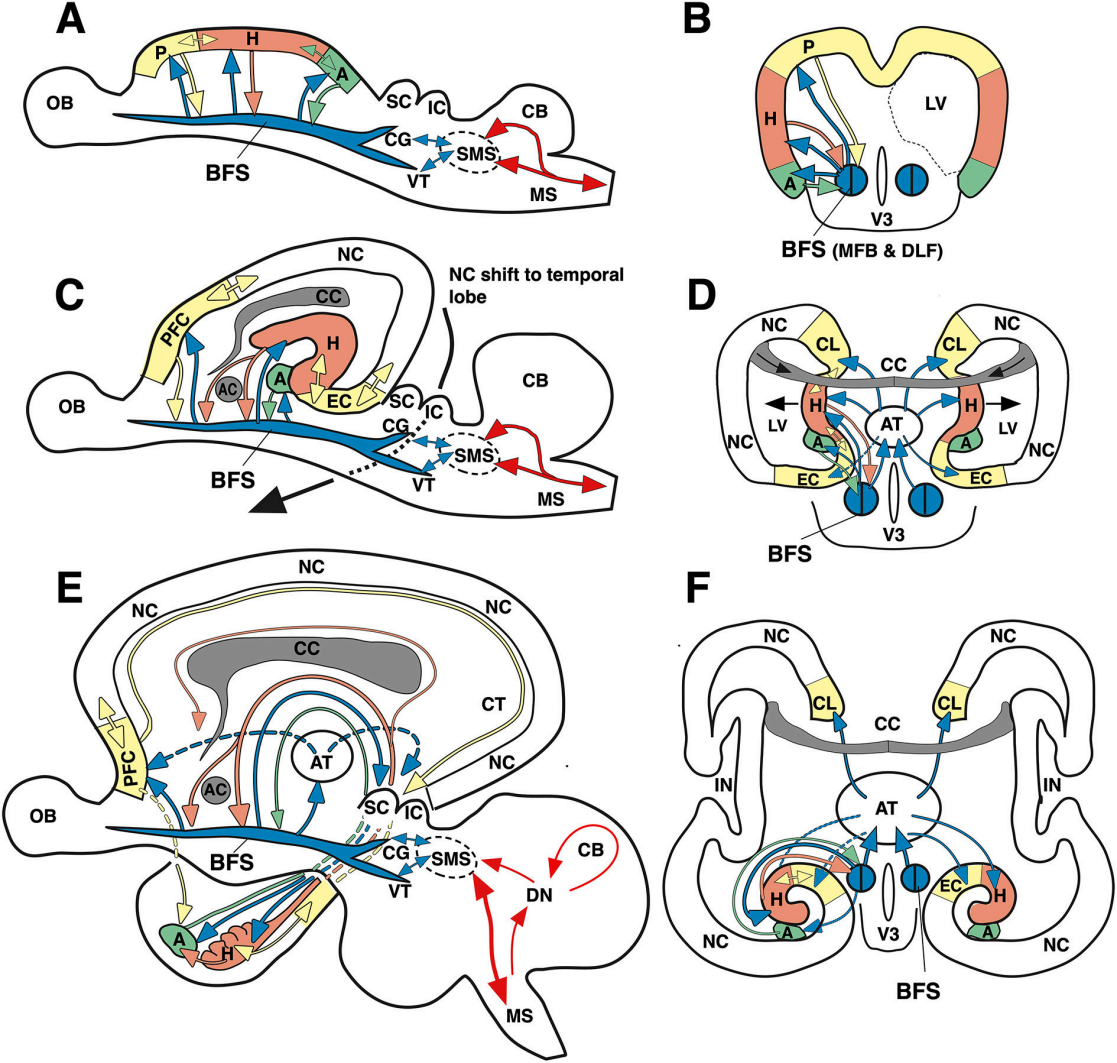
Simplified “evo-devo” sketch of the evolution of the fronto-limbic system in mammals and its connectivity with the BFS. The latter is formed by the rostral nuclei of the basal forebrain and the hypothalamic area, which are interconnected by the MFB (handling positive emotions and actions), with lower tail to the ventral mesencephalic area (VTA), and the DLF (handling chiefly negative emotions and avoidance actions), with the upper tail in the central gray region (CG). The latter structures connect to the supraspinal motors system (SMS) controlling the descending spinal motor system and its sensory feedbacks (but also priming upstreams the neocortex). **(A)** Lateral view of the evolutionary oldest arrangement in which the BFS establishes feedback loops with the overlaying archicortex (archipallium) for improving analysis of sensory inputs. The most rostral area (P) is analyzing olfactory inputs, becoming the prefrontal cortex (PFC), while a caudal area evolves for handling chemosensory and auditory inputs (becoming the later amygdaloid complex, A). An intermediate portion (H) becoming later the hippocampal formation analyzes activating inputs from the BSF and sends back facilitating or inhibitory feedback after having integrated inputs from both BFS and rostral and caudal archicortex. Note that the archicortex was stretched longitudinally here for better presentation of structures. **(B)** Cross-sectional (coronal view) of the primordial archicortex and the newly forming lateral ventricle (LV). V3 denotes the phylogenetically old third ventricle. The blue symbol for the BFS is divided indicating its composite nature. For a 3D view of the forebrain vesicles, see Supplementary Figure S3. **(C)** Lateral view of a rodent-type brain. The BSF and its caudal connections to the SMS remain unchanged, also its ascending tracts to the limbic cortex (prefrontal cortex, PFC, and entorhinal cortex, EC). However, the in-growth of the corpus callosum (CC) and the development of the neocortex (NC) push hippocampal formation, prefrontal and entorhinal cortex to the rim of the hemispheres (lat. “limbus”). The anterior commissure (AC) splits the hippocampal feedback into a pre-commissural and post-commissural fornix. **(D)** Cross-sectional view of the developing hemispheres shows the hippocampal formation bulging into the lateral ventricles, and the emergence of a major hub for the limbic cortex, the anterior (limbic) thalamus (AT) receiving unidirectional input from the BFS (through the mammillothalamic tract, MTT) thus amplifying its impact on the fronto-limbic system. **(E)** Lateral scheme of the human fronto-limbic system. The BFS and its ascending activating fiber tracts and their recurrent feedbacks input largely remain, including the hub function of the anterior thalamus (Coenen et al., 2012). The shift of amygdala and hippocampal formation into the depth of the temporal lobe results in elongation of the fornix (hypothalamo-hippocampal loops) and some hypothalamo-amygdalar loops, such as the stria terminalis. Entorhinal and prefrontal cortex remain connected through the cingular tract (CT) in the medial wall of the hemispheres. **(F)** Cross-sectional scheme of the human brain emphasizing the hub role of the anterior thalamus. For another view of human limbic circuitry, see Figure 2. CB, cerebellum, CL, cingular cortex. DN, deep cerebellar nuclei; IC, inferior colliculus; IN, insular cortex; OB, olfactory bulb, SC, superior colliculus.

Evolutionarily, it is conceivable that the hippocampal formation has developed as an associative structure straddling hypothalamus and basal forebrain and was then marginalized by the development of the neocortex and its direct sensory inputs as visualized in Figures 13A,B. Given the enormous density of intrahypothalamic connections needed to orchestrate physiology and behavior, it appears that during evolution the hypothalami of different vertebrate lines have added various types of gray matter to deal with sensory and proprioceptive information. In fishes, a lateral hypothalamic lobe is handling multisensory inputs and behavior (Schmidt, 2020), while in mammals, the rostral end of the telencephalon formed a vesicle whose walls formed a monolayered archicortex (the primordial limbic system). Its rostral parts (becoming prefrontal and entorhinal cortex) were handling olfactory input, its caudal parts (becoming the amygdaloid complex) were receiving chemical and auditory inputs, while the central portions (becoming the later hippocampus) had to balance these various inputs with proprioceptive (movement-related) signals generated by the SMS. However, in simplistic terms, this would imply that the hippocampus and its connected cortical structures would represent a kind of fine-tuning “slave processor” of basal forebrain and hypothalamus (Figures 13C–F), like the cerebellum for the motor system. This hypothesis conflicts with the usual view of the hypothalamus as downstream recipient of cognitive hippocampal processes and would require more upstream channels than usually assumed. The following section will thus focus on the hypothalamic projections to the hippocampus and limbic structures yet omitting upstream fibers in the septo-hippocampal system (Niewiadomska et al., 2009) and in the pre-commissural fornix (fibers rostral of the anterior commissure).

### A closer look at ascending hypothalamo–hippocampal connections

**Figure 14:**
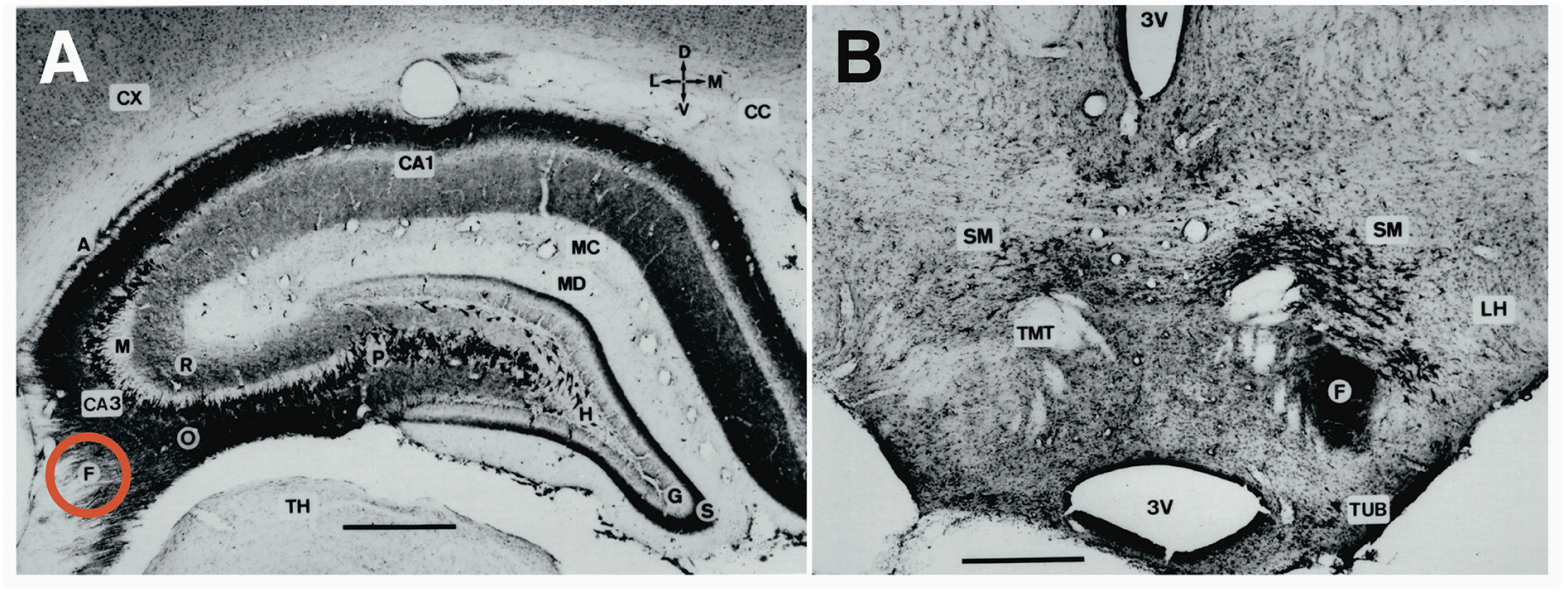
Retrograde and anterograde labeling of the contralateral dorsal rat hippocampus on a coronal section after placing a neuronal tracer (HRP) on a point where all fibers leave the hippocampus (the fimbria, F, red circle). Overlying structures had been removed by suction. **(A)** Darkly stained neurons indicate pyramidal cells (P) sending Schaffer collaterals to the other hippocampus, where they terminate in a darkly staining band below pyramidal cells (stratum oriens, O), or less densely above the pyramidal cells in stratum radiatum, R. Above the granule cell layer (G), afferent fibers of either commissural or subcortical origin form a dense band in the supragranular layer, S), a perfect location to control excitability of granule cells. **(B)** Coronal section through the posterior hypothalamus of the same rat showing strong retrograde labeling of numerous neurons in and around the supramammillary nucleus (SM, also labeled SUM elsewhere); demonstrating massive upstream connections to the hippocampus. Several dark-stained regions indicate descending fiber bundles to other hippocampal subregions, most massively in and near the fornix (F). The mammillothalamic tract (MTT) remained unstained, indicating lack of transneuronal anterograde transport. Other abbreviations: 3V, third ventricle; A, alveus (fiber mantle of hippocampus); CA3 and CA 4, hippocampal subfields; CC, Corpus callosum; CX, cortex; G, granule cell layer; LH, lateral hypothalamus; LM, stratum lacunosum-moleculare; M, suprapyramidal mossy fiber layer (also stratum lucidum); MC and MD, molecular layers; P or PYR, stratum pyramidale; S, supragranular layer; TH, thalamus; TMT, tractus mammillo-thalamicus (latin nomenclature for mammillothalamic tract); TUB, tuberal nuclei. Unpublished data by Lipp and Nauta (1984).

The presence of hypothalamic fibers ascending directly to the hippocampus has been repeatedly reported for squirrel and rhesus monkeys (DeVito and White, 1966; Poletti and Creswell, 1977; Senova et al., 2020), because it is relatively easy to cut or inject the fornix in primates as it is located under the corpus callosum. Labeling all fibers leaving or entering the rodent hippocampus through the fornix is more difficult because it would require filling the entire structure with neuronal tracer. Partial injections of horseradish peroxidase (HRP) into the hippocampus had revealed rather small numbers of retrogradely labeled neurons, chiefly in the dorsal supramammillary nucleus, SUM (Pasquier and Reinososuarez, 1978). Further studies of this region have shown that it is part of a widespread reciprocally connected network involving hippocampus and limbic cortices that appear topographically mapped into the hypothalamus (Pan and McNaughton, 2004). The SUM region is comparatively small but an old pilot study (Lipp and Nauta, 1984) had shown, by labeling the fimbria stump after suction of all overlying structures, that this projection appears massive (Figure 14 and legend for details). Preliminary quantification revealed about 20'000 ipsilateral labeled neurons that were sending ascending fibers to the hippocampus (Figure 14B). Given that the postcommissural fornix of rats contains some 40 to 50'000 fibers (Powell et al., 1957), this would imply that nearly half of the postcommissural fornix fibers are ascending from the SUM. Their endings in the hippocampus could not be determined with certainty (Figure 14A) but recent studies have shown that the area projects monosynaptically to the dentate gyrus, potentiating its output (Hashimotodani et al., 2018) and is possibly sending novelty signals (Chen et al., 2020). One may note that the dorsal SUM belongs to a hypothalamic cell group controlling central gray structures modulating species-specific defense behavior both to innate and conditioned threats (Wang et al., 2021; Bang et al., 2022) and so the concomitant alerting of the hippocampus by activation these nuclei is not surprising. Another candidate structure for showing links between hippocampus and species-specific behaviors is the VMH, whose electrical stimulation evokes aggressive-defensive behavior in many species (see The “old” brain and its connections). Recent studies have even identified “territorial” place cells in the mouse VMH, possibly established *via* a hippocampal loop (Krzywkowski et al., 2020).

An exclusively hypothalamic “powerhouse” is formed by the orexinergic neurons innervating diffusively the entire brain and co-regulating sleep–wakefulness, vigilance, feeding, energy, and neuroendocrine homeostasis, yet also partially motor behavior and reward mechanisms. Many of these fibers ascend through fornix and stria terminalis (Zhang et al., 2013). A similar cluster of activating histaminergic neurons with widespread efferent axons is the tuberomammillary nucleus (Blandina et al., 2012). Both nuclei are generally viewed as relay structures used by various other systems to tune specific brain functions, but the idea that they are a main tool of the hypothalamus to orchestrate the brain according to basic needs and motivation seems to be rare. A telling example is the role of the massive projection from the mammillary bodies (mammillo-thalamic tract, MTT, Figure 14B) to the anterior thalamus (AT), itself a hub innervating limbic cortex and the subiculum whose destruction causes cognitive, emotional, and memory deficiencies (Witter et al., 2017). Since the subiculum sends fiber through the fornix reaching the AT directly or indirectly through the mammillary bodies and the MTT, this so-called Papez loop is mostly viewed as a control system of the hippocampus by which navigational and memory-related information is passed through the hypothalamus for integrating basic motivational signals that will change the thalamo-cortical activation patterns of the AT (Aggleton et al., 2022). But the reverse view, namely that mammillary bodies send commands to the AT and limbic system, using the hippocampus for feedback information through the fornix, is apparently not much discussed, even though the mammillary bodies are known to drive head-direction cells and theta rhythms.

Taken together, an analysis of hypothalamic upstream connections strongly implies that the basic drivers controlling “higher-order” brain functions, including memory, motivations, emotions, and goal-directed behavior, are located along the longitudinal axis of the basal forebrain and interconnected by the MBF and DLF (see Figures 2, 13). These systems correct ongoing behavior by crude upstream signals that are then refined by cognitive and motor processing in associative cortices and hippocampus and fed back in condensed projections to these subcortical structures. Three simple arguments support such a view. (i) Evolutionarily, the basal forebrain structures appeared first. (ii) Destroying basal forebrain structures has mostly detrimental consequences for behavioral control, while loss of hippocampus and associative neocortex can be compensated over time, and (iii) the neuroanatomical analysis of cortico-subcortical loops shows that the focal control or set-points are subcortical.

Obviously, the bidirectional connections of the hippocampus with the BFS (together with the amygdala inputs and loops) are of special interest to translational research, specifically in the domain of deep brain stimulation (Senova et al., 2020), but the local density of subcortical target structures will pose problems due to co-stimulation of other loops.

## Viewing cognition through the lens of the motor system and hypothalamus

### The conventional scenario

The neocortex is traditionally viewed as the superior structure guiding behavior, a belief formulated in detail more than 200 years ago (Gall, 1822). For example, a widespread notion holds that the visual cortex perceives an interesting object and transmits somehow his discovery to the navigational brain parts, including hippocampus, that in turn tells the prefrontal cortex to make decisions and to take action by sending information to the midbrain and spinal cord instructing them what to do. The execution is then thought to be a largely automated process by the two control loops refining motor actions originating in the motor cortex as shown in Figure 2. In addition, the hemispheres appear to contain circuits labeled “social brain” or “visceral brain” or “computational brain” to name a few. Their common denominator is a top-to-bottom view as in Figures 2, 4, the various cortical systems busy in executing specific cognitive tasks pushing the motor system to act. Here, we present another perspective that appears helpful (at least to us) and might even resolve old riddles in the field.

### Mice think with their feet–the neocortex as a (dispensable) slave processor of the motor system

As formulated by one of the authors (D.P.W.) “mice think with their feet”. In analogy, there seem also many humans capable of thinking only while talking, as evidenced by countless hours spent in meetings with people arriving ill-prepared and grasping the topics for discussion only while speaking (Nauta W.H.J., oral communication, 1983). Thus, it appears that the activity of the neocortex is primed markedly by ongoing neuronal activity in midbrain and the basal forebrain. In computer terms, a small CPU can be equipped with two big graphic cards permitting complex computer games executed by simple inputs from a keyboard or a game console, ending with simple final output, “you lost”, so why not. The idea of the neocortex as a subordinate processor fed by mesencephalon, hypothalamus, and basal forebrain nuclei is not new, but apart from its logical appeal, it has received limited support from neuroscience. This is changing now by the advent of visualizing whole brain activity in various species (Kaplan and Zimmer, 2020) that seem to convey a similar message: ongoing motor activity is priming the activity of even sensory brain parts in different species, such as flies (Aimon et al., 2019), mice (Stringer et al., 2019; Parker et al., 2020), nematodes (Kaplan et al., 2020), and lampreys (Grillner et al., 2008).

### Turning the brain upside down

**Figure 15:**
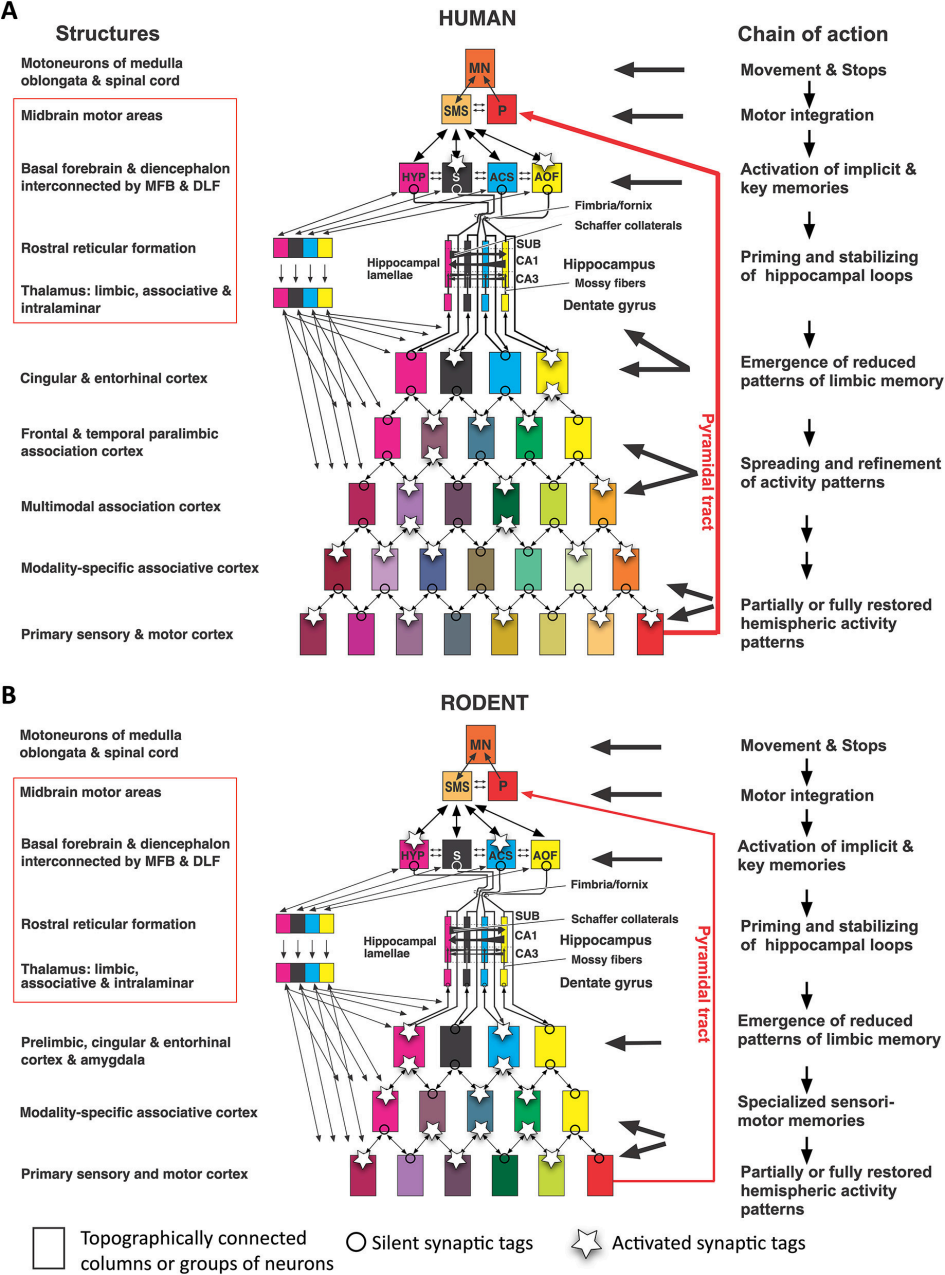
Reverse functional hierarchy corresponding to phylogenetic history. In both humans **(A)** and rodents **(B)**, the red-framed structures control movements based on primary motivations, simple directions, and ongoing active motor patterns. Cortical activity is primed by interactions between midbrain motor areas and by the structures of the basal forebrain interconnected by the medial forebrain bundle (MFB) orchestrating appetitive actions and more medially interconnected structures governing stress, fear, and escape reactions by the dorsal longitudinal fasciculus (DLF). In this view, the hippocampus is primarily fine-tuning the basal forebrain structures and setting synaptic tags there that stabilize lamellar loops but are also fed “downstream” to produce, jointly with thalamic and commissural inputs, increasingly complex activity patterns that eventually activate visual and auditory memories or dreams. Synaptic tags become activated by signals alerting the entire network, the stronger the signals, the more tags become activated, and the reconstructed memory pattern is more precise. In the case of few tags, the patterns fed in from the hippocampus may result in falsely reconstructed memories (Lipp, 2015). AOF, anterior olfactory nuclei; ACS, nucleus accumbens septi; CA1, hippocampal subfield; CA3, hippocampal subfield; HYP, hypothalamus; MFB, medial forebrain bundle; DLF, dorsal longitudinal fasciculus; MN, motoneurons; P, Pyramidal motor system; S, septal nuclei; SMS, supraspinal (mesencephalic) motor nuclei; SUB, subiculum.

By turning Figure 4 by 180°, Figure 15 shows the same components, but now the hierarchy is topped by the midbrain motor system (SMS), superior colliculus, hypothalamus, and the nuclei of the basal forebrain, forming together the very old core complex governing behavior in so many species. Simplistically, it illustrates that the information flow from midbrain reaches sensory areas through non-specific thalamic nuclei, while the hippocampus is now suddenly a structure that primarily receives patterned motivational information from the hypothalamus and sends back simple correcting signals, such as position and movement status, not unlike the cerebellum. In theory, it could be possible that the hypothalamus and basal forebrain are mapped topographically into the hippocampus, most rostrally the olfactory nuclei. But in parallel, the hippocampal loops also communicate with the entorhinal cortex from which the patterns are distributed tangentially to form increasingly complex configurations, perhaps generating complex memories, visions, or dreams, at least in humans. However, the visions will be structured by the parallel input from the SMS through non-specific thalamic nuclei ensuring that the patterns will develop along and align with motor signals generated in the midbrain. Three interesting points emerge from this concept.

1) The pyramidal tract in the human brain (Figure 15A) appears as a feedback loop rather than a commander. The relevant areas governing behavior and cognition, the phylogenetically old brain, are framed in red: midbrain motor areas, the basal forebrain nuclei, the rostral reticular formation, and the limbic and associative thalamus. The neocortex remains as a neuronal playground for free-floating patterns of excitation and various structured sensory inputs yet are much smaller in rodents (Figure 15B). An inhibitory clutch in the midbrain blocks unwanted selection of motor channels (Fraigne et al., 2015) in both species even though the “old brain” is feeding in motivational and ongoing motor activity patterns. The latter may be virtual while dreaming.
2) Downstream from thalamus, much of the neocortex is dispensable, at least for daily life and after some adaptation. The phylogenetically old brain structures are capable of learning, as shown for example by a rarely cited study by Huston and Borbely (1974) demonstrating that “thalamic” rats (with most forebrain structures removed) can be conditioned by rewarding hypothalamic stimulation when they adopted a particular posture. But there was no extinction of the response and they had to be primed by rewarding stimulation to adopt another one—hinting at a neocortical role of information selection and suppression. More recent work has shown that a two-year-old rat with massive hydrocephalus performed decently on a battery of behavioral tests, including spatial learning (Ferris et al., 2019). Later neuroanatomical analysis showed that it had a malformed hippocampus that was compressed into the lower hindbrain, together with hypothalamus, midbrain, and pons. Likewise, mice without cortex and hippocampus master many behavioral tasks, including predator avoidance (Turan, 2021).
3) Finally, it is well known but generally blanked out (or hotly disputed) by psychologists (Branwen, 2021) that there are recurrent observations of people living well without a cortex visible on brain scans (Lewin, 1980; Feuillet et al., 2007; Persad et al., 2021). The general cause is juvenile hydrocephalus wherein the growth of the ventricles is flattening and eventually suppressing the cortex and its fiber tracts. But what remains appear to be sufficient for enabling an existence as white-collar worker and family father in France (Feuillet et al., 2007), albeit handicapped by an IQ of 75, which, however, is still within the normal range and shared by some 12% of contemporaries with an intact brain. Notably, at the age of 44, his verbal IQ was 84, perhaps disguising his performance IQ of 70.

Obviously, mammals without a cortex and hippocampus can master their daily routines, but in the long run, they will be victims of natural selection. Whether the costly evolutionary addition of gray matter and comparatively more long fiber tracts in the human brain accounts for an average increase of 25% IQ is an intriguing pending question. For translational research, however, focusing on simple daily life and the minimal cerebral structures supporting it should be a guiding principle.

## Concluding perspectives

### Simple or complex tasks?

The main message of this article is to keep things simple (“mice think with their feet”) and to avoid explanations of rodent behavioral data in terms of human psychological concepts or theories. Given that the endpoint of any animal behavioral test is simple movements, translational researchers are on the safe side when opting for simple tests involving species-specific features. Usually, such tests are learned far more rapidly than observed in standard conditioning procedures (Rosenberg et al., 2021; Meister, 2022). Whenever possible, an initial focus on impaired naturalistic behaviors or even simple motor acts is justified, because restoring them by treatments will be closer to the causal roots. For example, if a drug aimed at curing hippocampal functions can re-establish nest-building and burrowing (Jirkof, 2014), it is translationally more valuable than a drug that reinstates water maze learning, because the latter is sensitive to many confounding mechanisms. Therefore, the treatment might cure (as so often in medicine) symptoms rather than causes. In any case, checking naturalistic behaviors is more efficient and faster than, for example, testing mice for visual pattern discrimination (Dickson and Mittleman, 2022).

Conversely, if the expected behavioral profile resulting from a mutation or a candidate drug is not clear, it helps to unravel the presence of deficits by subjecting the animals to tests limiting the degree of freedom in displaying behaviors, for example, avoidance learning, or to conduct forced tests like the water maze that challenge many brain systems beyond the hippocampus. An additional dissection of the symptoms is then mandatory, however. Whenever possible, animals should undergo different tests permitting analysis of individual scores.

A particular problem is the development of new tests and systems supposed to improve analysis. Few tests were accepted so rapidly as the water maze test already after its first presentation by Morris et al. (1982). Generally, new variants, procedures, and apparatus face problems: one is slow acceptance because established experience and data collections are jeopardized, especially in the pharmaceutical industry. Another one is the unwillingness of researchers to cross-validate their findings with other apparatus or protocols. A last and thorny one is standardization of tests, partly because of disagreement which ones to include or abandon, partly because the value of standardization itself is questioned (Würbel, 2002; Richter et al., 2009; Völkl et al., 2021). From the data presented here, it seems clear that standardization is overvalued and should not be sought in translational research. The lab of the authors has tested several thousand mice in highly standardized procedures, such as water maze learning, but the results obtained in totally non-standardized semi-naturalistic environments were identical, at least for hippocampal lesions and genetic malfunction, namely severe problems in reversal learning.

### Working with other species?

Inclusion of other species for behavioral testing can provide useful insights, for example, how wild species behave in the water maze task (Pleskacheva et al., 2000). After all, rats and mice represent only two cases out of about 5000 rodent species, and mice themselves are special because of their immediate motor reactions to threats. In terms of behavioral testing, this might require additional efforts, however. Yet, in some species such as dogs and primates, plain observation of social interactions and depressive behavior might provide important insights. For example, studies in dogs have shown that interconnected parts of amygdala and hypothalamus might form two *tonically* active yet antagonistic loop systems regulating socio-positive and socio-negative emotions and behaviors whose balance can be regulated by lesions of different amygdala structures (Fonberg, 1973). Such patterns can be easily observed in dogs but are difficult to recognize in rodents. Likewise, Patas monkeys (*Erythrocebus patas*) are gregarious during the daytime but spend the night on separate trees whenever possible (Hall, 1966; Gron, 2006). Such circadian oscillation between socio-positive and socio-negative behavior might deserve telemetric investigation of electrical activity in amygdala and hypothalamus.

### Which brain systems to investigate?

#### Translational behavioral analysis should not confound system properties of networks with physiological properties of neurons

Given our obvious penchant toward neuroethological and observational approaches, we recommend checking a review by Vanderwolf and Cain (1994). It disentangles the neurobiological meanings of memory and learning, arguing that research in learning and memory should be pursued by biological studies of animal behavior, combined with a cellular/molecular approach in neuronal function yet without equating the two approaches. The last two sections are of special interest to translational behavioral research: (1) learning in real-life situations is likely to involve the participation of multiple brain structures and their connectivity. Thus, investigations should not single out structures of actual interest, and behavioral assessment should not only use conventional maze learning and sensory discrimination tasks, but also natural behaviors related to parental care, dominance, or territoriality. (2) The translational study of neurodegenerative diseases such as Alzheimer's disease (AD) appears heavily biased by studying hippocampal memory deficits yet paying less attention to socially and clinically more relevant behavioral changes, such as irritability, phases of locomotor hyperactivity, and showing dangerous behaviors. We agree that such commonalities are likely to be shared by mice and men and should move into the focus of neurobehavioral translational research but suggest being open for new hypotheses.

#### Neocortex or subcortical structures?

In terms of specific brain systems for translational research in the domain of psychiatry and neuropsychology, concentrating on phylogenetically old systems such as the hypothalamus appear appropriate when emotions or depression are involved. The wealth of data from hypothalamic stimulation in different species, including rats and mice, suggests that this brain region might be a preferable target for deep brain stimulation (DBS) involving fornix and amygdala projections (Senova et al., 2020), and possibly also vagal afferent and efferent fibers (Breit et al., 2018). It should be considered, however, that the mode of action of DBS for the treatment of depressions is poorly known (Ramasubbu et al., 2018). Given that the well-known improving effects of DBS in Parkinson's patients can also be obtained by MRI-guided ultrasound surgery (Gallay et al., 2018), one might consider whether a lesion approach in laboratory animals might be a cheaper strategy for studying and evaluating psychosurgery.

If the problems appear to be autism or cortical malfunction, the theoretically most efficient focus would be the inhibitory systems of the thalamus that tune ascending channels to the cortex. However, they are not easily targeted. The approach by Simmons et al. (2021) to focus on simple sensory channels for discovering intrathalamic malfunction could thus be helpful.

#### The hippocampus as a sensor of malaise and wellbeing?

While the role of the hippocampus in regulating the stress axis through mineralo- and glucocorticoid receptors is well established, it should also be considered that the hippocampal formation harbors the highest density of some 250 murine endocrine receptors for blood-borne ligands, and that these receptors show a spatially segregated expression in the hippocampal formation (Lathe, 2001; Lathe et al., 2020). These findings hint at hitherto unrecognized organizing functions of the hippocampus that could be of relevance to translational research. To our knowledge, it is poorly understood why being sick shuts down activity levels permitting better recovery but also reducing cognitive abilities. Lathe's hypothesis implies that an evolutionarily primeval chemosensory function of the hippocampus was enteroceptive, permitting this structure to coordinate body physiology, hormone levels, emotions, and synaptic memory processes in spatially separated channels.

A more concrete translational approach might be malfunctions of the hippocampal mossy fiber system (see section A neglected feature for translational research: mossy fiber distribution and behavior). Pathological sprouting within the mossy fiber distribution was observed after castration in rats (Skucas et al., 2013; Scharfman and MacLusky, 2014; Mendell et al., 2017). It might also occur after prostatectomy in men because this is often followed by “chemical castration” such as androgen deprivation therapy (ADT) lowering testosterone levels for inhibiting the growth of cancer cells. Interestingly, the human hippocampus shows about the same density of androgen receptor mRNA as the prostate tissue itself (Beyenburg et al., 2000), raising the question of whether ADT in men might also entail mossy fiber sprouting and hippocampal malfunction. Problems with short-term memory are frequent after ADT (Alibhai et al., 2010; Kluger et al., 2020). For example, a patient intends to go to the garage but ends up in front of the refrigerator having forgotten that he was hungry. In the scenario given in Figure 14, the initial impulse would originate in the hypothalamus, then being fed to the (impaired) hippocampus for propagation to the limbic cortex but will not be integrated with the ongoing motor activity, faking a cognitive short-term memory problem.

### Classic behavioral test batteries or home-cage tests?

For translational research, assessment of behavior in animals, TV-controlled and electronic surveillance by implanted RFID chips in home cages is preferable as this fits the ARRIVE guidelines for sustainable research data much better (du Sert et al., 2020), while the use of large test batteries, complex operant conditioning or complex mazes would seem less important because they lower reproducibility of behavioral studies—a most important point for acceptance in science (Ioannidis, 2014). This holds specifically for mice. For one, an anxiety-inducing experimenter effect is always present (Nigri et al., 2022), unless the mice climb on the hand of the scientist to undergo testing willingly. The second reason is that systems such as Phenotyper^®^ or IntelliCage^®^ permit monitoring behavior over days or weeks, and free humans for other tasks. In doing so, they can detect many mutations or dysfunctions just by patterns of spontaneous activity (Dell'Omo et al., 2002; Vannoni et al., 2014) and can measure effortlessly behavioral flexibility and even rule-learning (Endo et al., 2011). Another ethologically oriented multi-individual home-cage system is the EcoHab (Puscian et al., 2016), which is particularly well-suited to study automatically social preferences and olfactory discrimination of familiar and unfamiliar conspecifics—behaviors essential for studying autism-like traits in mice.

Lastly, focusing on simple behaviors and responses will also facilitate the use of machine learning recognizing behavior patterns eluding human observation. However, this should occur in varied environments to avoid machine-learning of apparatus- or situation-specific properties and must be cross-validated. Admittedly, this perspective is intellectually less challenging than traditional behavioral research, but translational approaches must focus on a given human problem regardless of the preferences of the investigator.

## Author's note

Translational research in behavioral and psychiatric neuroscience faces difficult problems. While neurobehavioral scientists in basic research are free to use species, concepts, methods, and approaches, translational scientists are constrained as they must read and interpret animal movements in terms of human psychological processes. This article identifies and illustrates practical problems, different theoretical views, and hidden pitfalls in interpreting animal behavior in human psychological terms. The main translational problems are inadequate knowledge in comparative neuroanatomy and ecology, anthropomorphizing animals (especially mice), and conceptual flaws in interpreting behavioral changes after treatments. This article suggests a neuroethological approach emphasizing that neocortical selection of observable motor patterns is primed primarily by ongoing activity of both hypothalamus and the midbrain supraspinal motor system. In practice, this means that translational research should focus on analysis and opportunities for intervention in these structures, assessing behavioral changes by complementary automated supervision and machine learning.

## Author contributions

H-PL wrote the major review part. DW contributed graphs and statistical analysis. Both authors have contributed equally to many of the reviewed publications.

## Funding

The review part was supported by intramural funds of the University of Zürich to H-PL (R-42900-01-01) while the unpublished data parts were obtained with the support of the following grants: Swiss National Science Foundation SNFS 037497 (Genetical variability of brain and behavior in near-natural settings), SNFS 046691 Brain, behavior and ecology: experimental natural selection of brain traits, and SNFS 057139 Micro- and macro-evolution of the hippocampal mossy fiber system to H-PL and DW. Further support was provided by SNFS 054184 (The role of neuroserpin and extracellular proteolysis in learning and memory) to DW, National Competence Center for Neural Plasticity and Repair CTE2 to H-PL and DW, FP6 STREP/SME 037965 (Intellimaze) to H-PL and DW, European Consortium on Synaptic Protein Networks in Neurological and Psychiatric seases, HEALTH-F2-2009-241498 (EUROSPIN) to H-PL and DW, EMDO Foundation Zürich, (The role of neuroserpin in the regulation of learning and anxiety related behavior) and Zürich Center for Integrative Human Physiology (Enhanced cognitive functioning and memory by hypoxia and erythropoietin) to DW, and PSRP-2010-2010 (Polish-Swiss-Research Programme) to H-PL.

## Conflict of interest

The authors declare that the research was conducted in the absence of any commercial or financial relationships that could be construed as a potential conflict of interest.

## Publisher's note

All claims expressed in this article are solely those of the authors and do not necessarily represent those of their affiliated organizations, or those of the publisher, the editors and the reviewers. Any product that may be evaluated in this article, or claim that may be made by its manufacturer, is not guaranteed or endorsed by the publisher.
