# Supplementary material for: Behavior is movement only but how to interpret it? Problems and pitfalls in translational neuroscience—a 40-year experience
Source: Front Behav Neurosci. 2022 Oct 5;16:958067. doi: 10.3389/fnbeh.2022.958067 (PMC9623569; doi:10.3389/fnbeh.2022.958067)
Supplement: Supplementary file 1 [file Data_Sheet_1.PDF]

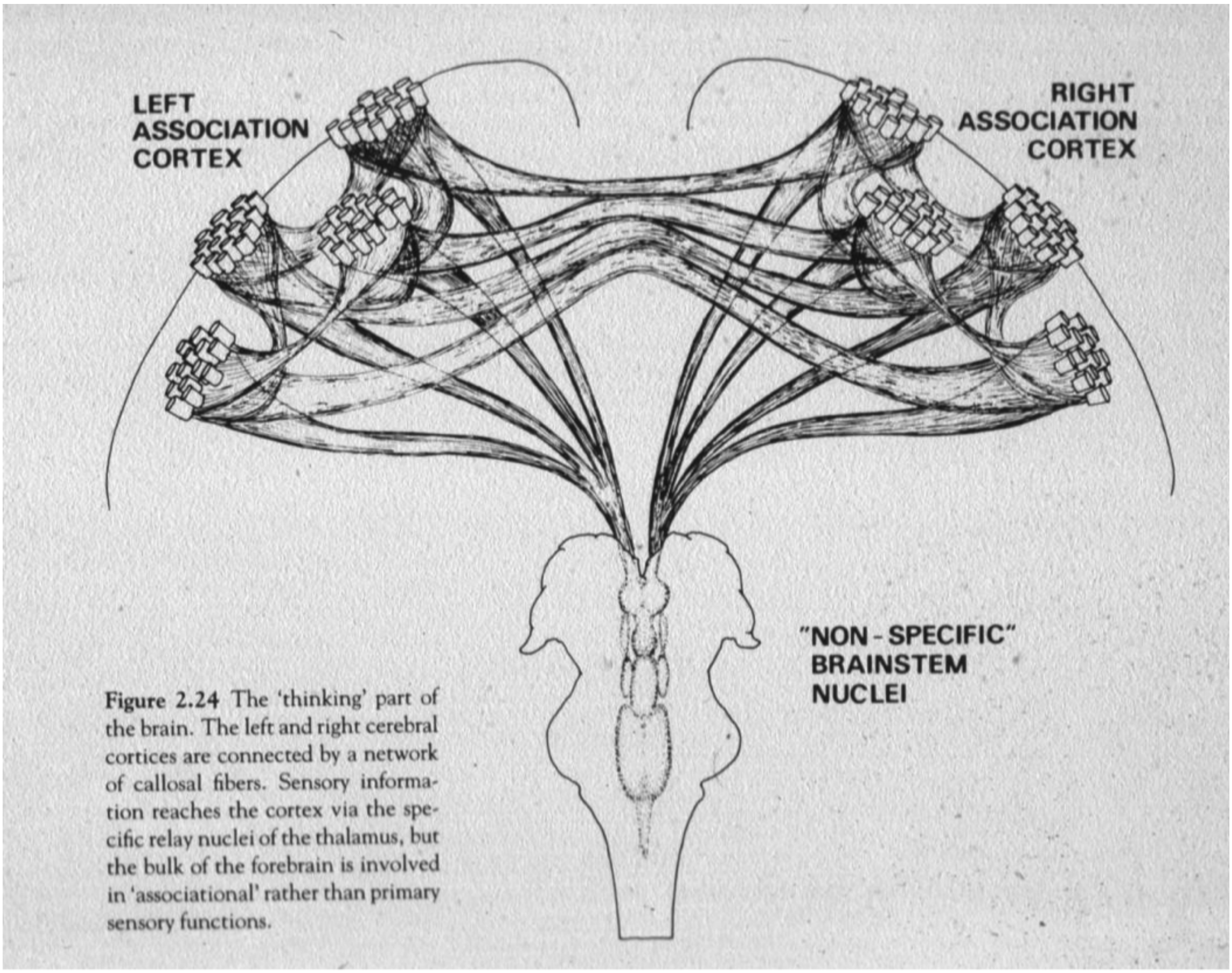

Fig. S1. Artistic 3D image of the connections of subcortical structures as specified in Figure 2. The figure was published first by Cook (1986) and reproduced with permission from Routledge and CRC Press.
