# Supplementary material for: Behavior is movement only but how to interpret it? Problems and pitfalls in translational neuroscience—a 40-year experience
Source: Front Behav Neurosci. 2022 Oct 5;16:958067. doi: 10.3389/fnbeh.2022.958067 (PMC9623569; doi:10.3389/fnbeh.2022.958067)
Supplement: Supplementary file 2 [file Data_Sheet_2.PDF]

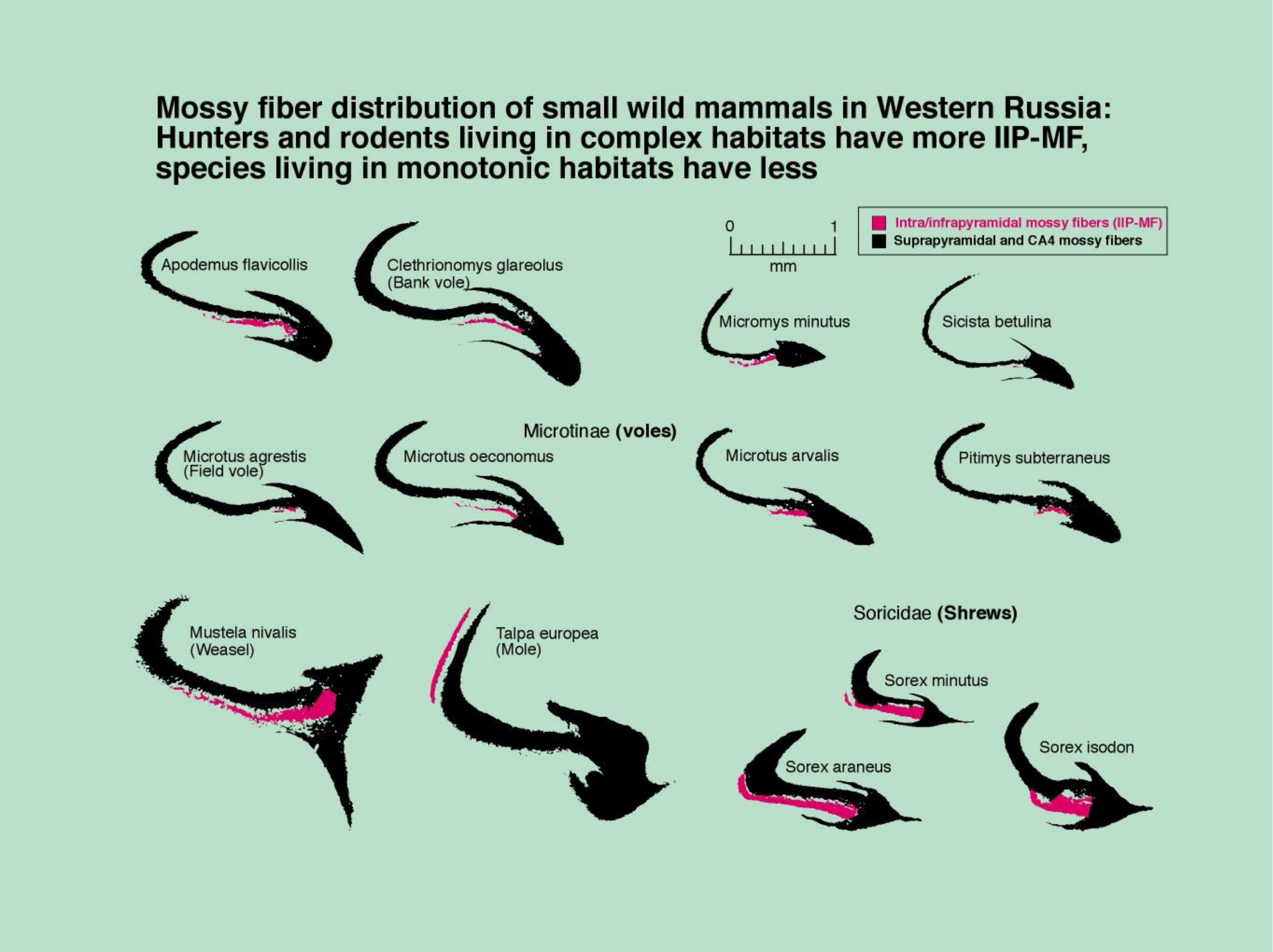

Fig. S2 Mossy fiber patterns of wild-living species in Russia. Animals were collected by Irmgard Amrein and Natasha Bologova
