# Supplementary material for: Behavior is movement only but how to interpret it? Problems and pitfalls in translational neuroscience—a 40-year experience
Source: Front Behav Neurosci. 2022 Oct 5;16:958067. doi: 10.3389/fnbeh.2022.958067 (PMC9623569; doi:10.3389/fnbeh.2022.958067)
Supplement: Supplementary file 3 [file Data_Sheet_3.PDF]

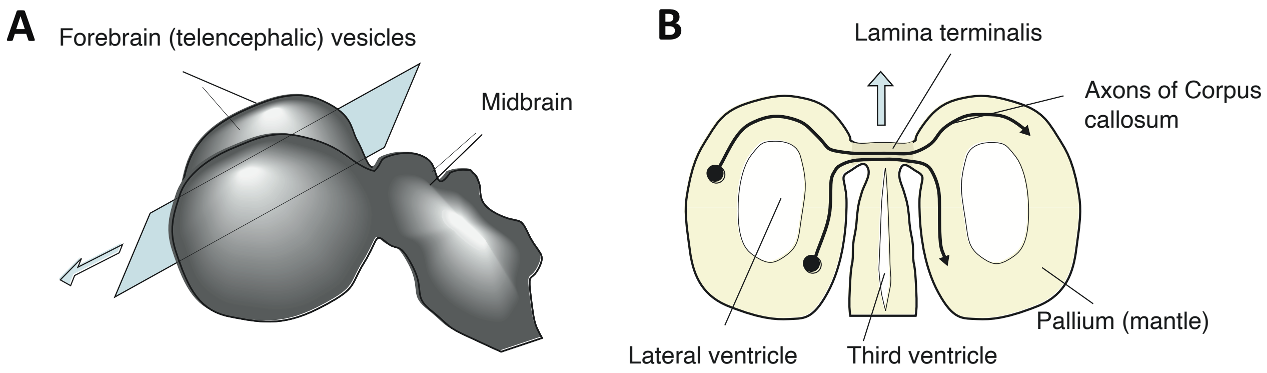

Fig. S3.

A) Embryological development of the mammalian cortex from two vesicles at the rostral end of the basal forebrain (the telencephalon).

B) Horizontal section plane. The initial layer of neurons and glial cells is referred to as pallium. Neurons and glial cells are produced in the cellular lining of the newly formed lateral ventricles, neurons migrating towards the surface. In the course of evolution, the first type of cortex was a one-layered archicortex typical for dentate gyrus and hippocampus. The corpus callosum develops from axons crossing the midline in a thin layer of glial tissue (lamina terminalis) .
